# Methylation-mediated LncRNA CRAT40 promotes colorectal cancer progression by recruiting YBX1 to initiate RelA transcription

**DOI:** 10.7150/ijbs.105629

**Published:** 2025-07-25

**Authors:** Qing Lu, Xiuhe Lv, Jin Wang, Bihan Xia, Hailin Yan, Zhu Wang, Jinlin Yang

**Affiliations:** 1 Department of Gastroenterology and Hepatology, West China Hospital of Sichuan University, Chengdu 610041, Sichuan, China.; 2 Department of Gastroenterology and Hepatology, Sichuan University-Oxford University Huaxi Gastrointestinal Cancer Centre, West China Hospital of Sichuan University, Chengdu 610041, Sichuan, China.

**Keywords:** colorectal cancer, long non-coding RNA, YBX1, METTL3

## Abstract

Colorectal cancer (CRC) remains a leading cause of cancer-related mortality worldwide. Long noncoding RNAs (lncRNAs) have emerged as crucial regulators in the initiation and progression of various malignancies, including CRC. In this study, we found that lnc-CRAT40 was upregulated in CRC and associated with poor prognosis following CRC resection. Functional assays revealed that elevated lnc-CRAT40 expression promotes tumor cell proliferation and metastasis both in vitro and in vivo. The modification of N6-methyladenosine, driven by METTL3, was essential for the stability of lnc-CRAT40, which may partially contribute to the upregulation of lnc-CRAT40. Mechanistically, lnc-CRAT40 directly interacted with Y-box binding protein 1 (YBX1) and recruits it to the RelA promoter, thereby activating NF-κB signaling, which in turn drives CRC proliferation and metastatic potential. These findings provide novel insights into the molecular mechanisms underlying CRC progression and highlight lnc-CRAT40 as a potential prognostic biomarker and therapeutic target.

## Introduction

Colorectal cancer (CRC) is among the most common malignancies worldwide, with rising incidence and mortality rates^1^. Despite significant advancements in therapeutic approaches, including surgery, radiotherapy, and chemotherapy, the overall prognosis of CRC remains poor, largely due to late diagnosis, recurrence, and metastasis^2^. As such, it is imperative to further elucidate the molecular mechanisms underlying CRC initiation and progression to identify early diagnostic biomarkers and novel therapeutic targets.

Recent advances in molecular biology have unveiled the intricate regulatory networks that drive cancer, with long non-coding RNAs (lncRNAs) emerging as pivotal regulatory molecules^3^. LncRNAs, a class of non-coding RNAs longer than 200 nucleotides, do not encode proteins normally but have gained prominence as critical contributors to oncogenic processes. Their altered expression and activity have been convincingly validated by functional studies, highlighting their significance in tumor biology. In CRC, specific lncRNAs have been identified as key regulators of tumor initiation, progression, metastasis, and chemoresistance^4^-^6^.

The subcellular localization of lncRNAs is essential to their regulatory function. Many lncRNAs are localized in the nucleus, where they interact with proteins, RNA, and DNA, modulating various gene regulatory mechanisms^7^. LncRNAs binding to proteins, particularly interactions with RNA-binding proteins (RBPs), regulate the onset and progression of CRC by influencing protein post-transcriptional modifications, stability, or subcellular localization^8^, ^9^. In CRC, these interactions significantly impact tumor biology. Notably, the interaction between lncRNAs and Y-box binding protein 1 (YBX1) plays a critical role in regulating CRC development and progression. LncRNAs modulate CRC pathogenesis by affecting YBX1 stability, nuclear localization, and its interaction with gene regulatory elements, thereby influencing gene transcription and tumor progression^10^, ^11^.

N6-methyladenosine (m^6^A) is one of the most prevalent internal RNA modifications, presents in both mRNA and non-coding RNA in eukaryotes^12^-^14^. As a dynamic and reversible modification, m^6^A is regulated by methyltransferases (“writers”), demethylases (“erasers”), and proteins that recognize and bind to m^6^A sites (“readers”)^15^. In CRC, m^6^A regulates gene expression, RNA metabolism, and signaling pathways, influencing the proliferation, invasion, and metastasis of cancer cells. Furthermore, m^6^A plays a critical role in regulating the stability of lncRNA by controlling its degradation and translation efficiency. METTL3, the key m^6^A writer, catalyzes the addition of methyl groups to specific RNA sites^16^. Through binding with m^6^A reader proteins such as IGF2BP3 or YTHDF2^10^, ^17^, ^18^, it recognizes the m^6^A modification sites on lncRNA and regulates their stability, thus impacting cancer progression.

In this study, we investigated the role of lncRNAs in CRC progression and delineated their mechanistic pathways. We identified a specific lncRNA, CRAT40, that is significantly upregulated in CRC tissues and associated with poor patient prognosis. Further analysis revealed that this upregulation is driven by METTL3-mediated m^6^A modifications, which stabilize the lnc-CRAT40 transcript. Our in vitro and in vivo studies demonstrated that lnc-CRAT40 promotes CRC cell proliferation and metastasis. Mechanistically, lnc-CRAT40 interacts directly with YBX1, modulating the transcription of the RleA gene and promoting CRC progression. These findings suggest that lnc-CRAT40 may serve as a potential biomarker and therapeutic target in CRC.

## Materials and Methods

### Subjects and specimens

Colorectal cancer (CRC) tissues and paired adjacent non-tumorous mucosa were collected from patients who underwent surgical resection at West China Hospital of Sichuan University. All specimens were snap-frozen in liquid nitrogen within 10 min and stored at -80 °C until RNA extraction. Overall survival (OS) was calculated from the date of surgery to death or last follow-up. Written informed consent was obtained from every participant, and the study was approved by the Institutional Ethics Committee of West China Hospital of Sichuan University. Clinicopathological features are summarized in Table 1.

### Cell culture

Human CRC cell lines SW480, HCT15, and LS174T, the normal colonic epithelial cell line HCoEpiC, and HEK293T cells were purchased from the Chinese Academy of Sciences Cell Bank (Shanghai, China). Cells were cultured in Dulbecco's Modified Eagle's Medium (DMEM; Cat# 11965092, Gibco, USA) supplemented with 10 % fetal bovine serum (FBS; Cat# ST30-3302P, PAN-Biotech, Germany) and 1 % penicillin-streptomycin (Cat# 15070063, Gibco, USA) at 37 °C in a humidified incubator with 5 % CO₂.

### Plasmid construction and transfection

CRAT40 knockout was achieved with a dual-sgRNA CRISPR/Cas9 strategy. Two target sequences (Table S2) were cloned into pX330 vector. GFP-positive cells were isolated by fluorescence-activated cell sorting. For overexpression, full-length lnc-CRAT40 was inserted into pcDNA3.1(+); full-length YBX1 cDNA was sub-cloned into pLVX-ZsGreen (FitGene, China). METTL3 shRNA oligonucleotides were ligated into pLKO.1-puro (FitGene, China). All siRNAs and antisense oligonucleotides (ASOs) were synthesized by RiboBio (Guangzhou, China) and are listed in Table S2. Transfections were performed with Lipofectamine 3000 (Cat# L3000001, Thermo Fisher Scientific, USA) according to the manufacturer' protocol.

### RNA extraction and quantitative RT-PCR

Total RNA was extracted using TRIzol Reagent (Cat# 15596018, Invitrogen, USA). cDNA synthesis was performed with PrimeScript™ RT Kit (Takara, Japan). Quantitative PCR was conducted on a CFX96 platform (Bio-Rad, USA) using iQ™ SYBR® Green Supermix (Cat# 170-8880, Bio-Rad, USA). Relative expression was determined by the 2^-ΔΔCt^ method with β-Actin (mRNA) or U6 (lncRNA) as internal controls.

### Cell proliferation assay

Cell viability was evaluated with Cell Counting Kit-8 (CCK-8; Cat# M4839, AbMole, USA). Ten microlitres of CCK-8 solution was added to each well of 96-well plates at indicated time points, incubated for 2 h at 37 °C, and absorbance was read at 450 nm.

### Colony formation assay

Approximately 1000 cells were seeded per 6-well plate and cultured for 14 d. Colonies were fixed in 4 % paraformaldehyde (Cat# BL539A, Biosharp, China) for 20 min, stained with 0.1 % crystal violet (Cat# C0121, Beyotime, China) for 30 min, washed, air-dried, and counted under a light microscope.

### Cell cycle analysis

Cells were fixed in 70 % ethanol at 4 °C overnight, stained with propidium iodide using a Cell Cycle Assay Kit (Cat# KTA2020, Abbkine, China), and analysed by flow cytometry.

### Wound-healing assay

A linear wound was created using a two-chamber culture insert (China). 1.0-1.5 × 10⁵ cells were seeded in each side of the insert in a 6-well plate and allowed to adhere for 12 hours. The insert was then removed. Cells were rinsed with PBS, cultured in serum-free DMEM, and imaged at 0, 24, and 48 h.

### Transwell migration and invasion assays

Cell migration and invasion were assessed using 24-well Transwell chambers (8 µm pore size; Cat# 3422, Corning, USA). For invasion, inserts were pre-coated with Matrigel (1:8 dilution; 100 µL, Corning, USA). Cells (2 × 10⁴) in serum-free DMEM were seeded in the upper chamber; medium containing 10 % FBS was added below. After 48 h, cells on the lower membrane were fixed, stained with crystal violet.

### Subcellular fractionation assay

Cells was added 200μL Lysis Buffer (5mM Tris-HCl (pH = 7.5), 1.5 mM MgCl2, 140 mM NaCl, 10% TritonX-100), resuspended and centrifuged at 300g for 2min at 4℃. The supernatant was the cytoplasm. The above precipitate was added with 400μL lysis Buffer, blown three times, and centrifuged at 300g for 2min at 4℃. The purified RNA was analyzed by qRT-PCR. The supernatant was discarded and repeated twice. The remaining was nucleus. U6 and GAPDH were used as nuclear control and cytoplasmic control, respectively.

### Animal experiments

All animal procedures conformed to the NIH Guide for the Care and Use of Laboratory Animals and were approved by the Laboratory Animal Ethics Committee of West China Hospital (approval no. 20220124001). Female BALB/c nude mice (4-6 weeks) were housed under SPF conditions. For subcutaneous tumors, 2 × 10⁶ HCT15 cells were injected into the flank; tumor volume was measured every 5 d. For liver metastasis, 2 × 10⁶ cells were injected into the splenic capsule. Eight weeks later mice were euthanised; livers and primary tumors were excised, weighed, and processed for histology.

### Immunohistochemistry

Tissues were sectioned at 4 µm, deparaffinised with xylene, rehydrated, and endogenous peroxidase was blocked with 3 % H₂O₂. After microwave antigen retrieval in citrate buffer, sections were blocked with 5 % BSA and incubated overnight at 4 °C with primary antibodies. HRP-conjugated secondary antibodies were applied for 1 h. Signals were developed with DAB and counterstained with haematoxylin.

### RNA pull-down and mass spectrometry

Cell lysates were incubated with biotin-labeled RNA probes synthesized by RiboBio (Guangzhou, China), followed by pull-down with Dynabeads™ MyOne™ streptavidin T1 (Cat#65604D, Thermo Fisher Scientific, USA) according to the manufacturer's protocol. The bound proteins were eluted and subjected to mass spectrometry analysis for identification.

### RNA immunoprecipitation (RIP)

Approximately 1 × 10^7^ cells were lysed in RIP lysis buffer and immunoprecipitation was carried out by incubating the lysate with magnetic bead-antibody complexes at 4 °C overnight. RNA was purified by phenol:chloroform:isoamylalcohol. RNAs combined with protein were isolated and then detected by PCR.

### Fluorescence *in situ* hybridization

Biotin-labeled DNA probes were synthesized by RiboBio, and the hybridization was performed using a FISH Kit (RiboBio, China) following the manufacturer's protocol. Nuclear staining was carried out with DAPI. Fluorescence signals were visualized and captured using a fluorescence microscope.

### Immunofluorescence

Cells were fixed with 4% paraformaldehyde, permeabilized with Triton X-100, and incubated with primary antibodies at 4 °C overnight. The following day, cells were washed twice and incubated with fluorophore-conjugated secondary antibodies. Nuclear staining was performed using DAPI. Stained cells were observed and imaged using a fluorescence microscope.

### Protein extraction and western blotting

Total protein was extracted using IP/WB Lysis Buffer (Cat# P0013, Beyotime, China) supplemented with protease/phosphatase inhibitors. Equal protein amounts were separated by 10 % SDS-PAGE (Cat# PG112, Epizyme, China), transferred to PVDF membranes (0.2 μm pore size), blocked in 5 % skimmed milk, and incubated overnight at 4 °C with primary antibodies (Table S5). HRP-labelled secondary antibodies were applied for 1 h, and bands were visualised with Pierce™ ECL substrate (Cat# 32209, Thermo Fisher Scientific, USA).

### Chromatin isolation by RNA purification (ChIRP)

The ChIRP assay was conducted using the Magna ChIRP RNA Interactome Kit (Cat#17-10494, Millipore, USA) according to the manufacturer's instructions. Briefly, 1 × 10^7^ cells were lysed in complete lysis buffer, and chromatin was sheared to 200-500 bp fragments by sonication. Lysates were then incubated with biotin-labeled probes targeting lnc-CRAT40 or control probes. Streptavidin magnetic beads were used to capture the probe-chromatin complexes. The purified DNA was subjected to qRT-PCR analysis.

### Chromatin immunoprecipitation (ChIP)

ChIP assays were performed using the SimpleChIP® Enzymatic Chromatin IP Kit (Cat#9003S, CST, USA). Briefly, cells were cross-linked with 1% formaldehyde, followed by quenching with 0.125 M glycine. Cells were lysed and sonicated to produce soluble chromatin fragments of 200-1000 bp. Pre-cleared chromatin was incubated overnight at 4 °C with antibodies against IgG or YBX1, followed by capture with Protein A/G Magnetic Beads (Cat#HY-K0202, MedChemExpress, USA). After extensive washing, the bound DNA was eluted and analyzed by qRT-PCR using ChIP-specific primers. Primer sequences are detailed in Table S2.

### RNA stability assay

CRC cells were treated with 15 µg/mL actinomycin D (Cat#50-76-0, MedChemExpress, USA) to inhibit transcription. Cells were then collected at various time intervals. The expression levels of lnc-CRAT40 were assessed using qRT-PCR.

### Methylated RIP (MeRIP)-qPCR

Methylated RNA immunoprecipitation (MeRIP) was performed according to the manufacturer's instructions using the Magna MeRIP m6A Kit (Millipore, Germany). The m6A-specific antibody was pre-incubated in IP buffer and conjugated with Magna ChIP Protein A/G Magnetic Beads at room temperature for 1 hour. The fragmented mRNA was then added to the antibody-bead complex and incubated overnight at 4 °C. Enrichment of m^6^A-modified RNA was subsequently analyzed by qRT-PCR.

### Dual-luciferase reporter assay

HEK293T cells were co-transfected with pGL3-firefly luciferase reporter containing the RELA promoter (2 kb), pRL-TK Renilla luciferase control, and indicated expression vectors using Lipofectamine 3000. After 48 hours, firefly and Renilla luciferase activities were sequentially measured using commercial substrates and a multimode microplate reader. Relative luciferase activity was expressed as Firefly/Renilla.

### Statistical analyses

Continuous variables were presented as means ± standard deviation (SD) or median (interquartile range), and compared using the Student's t-test or Mann-Whitney U-test. Categorical variables were reported as numbers and percentages, and compared using the Chi-square test. Survival curves were analyzed using the Kaplan-Meier method and log-rank test. Logistic regression analysis was used for multivariate correlation analysis. All statistical analyses were performed using IBM SPSS (SPSS, Chicago, IL, USA) and GraphPad Prism 9.0.0 (GraphPad Software, Inc.). Statistical significance is indicated as *P<0.05, **P<0.01, ***P<0.001 and ****P<0.0001.

## Results

### Lnc-CRAT40 is upregulated in CRC and correlates with poor prognosis

**Figure 1:**
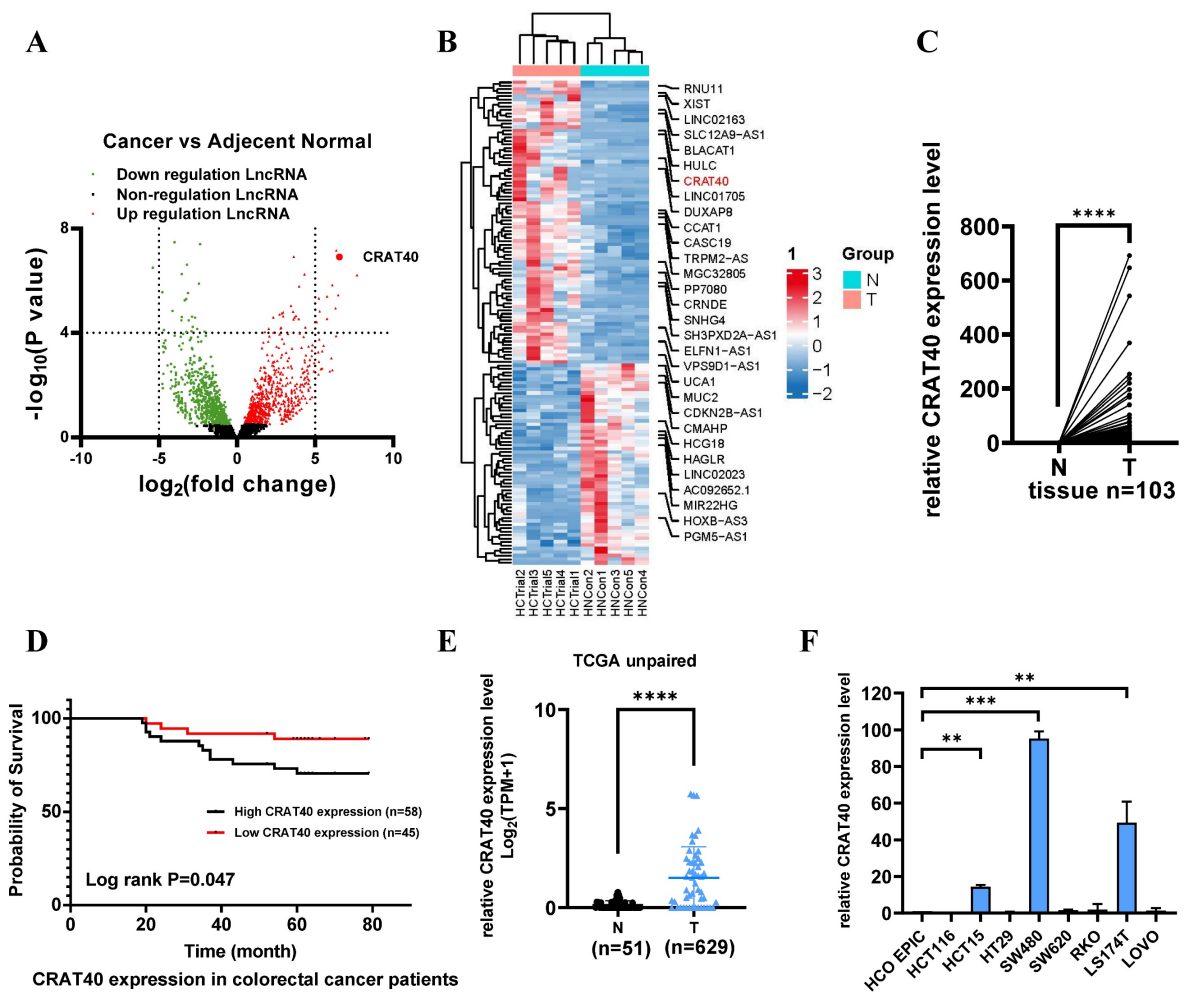
** LncRNA CRAT40 is upregulated in CRC and associated with poor prognosis. A.** Volcano plot of RNA-seq results showing differentially expressed lncRNAs in 5 pairs of CRC tumor and adjacent normal tissues. Each dot represents a gene; red dots indicate significantly upregulated lncRNAs, green dots indicate significantly downregulated ones. **B.** Heatmap of selected differentially expressed lncRNAs, with lnc-CRAT40 highlighted in red. **C.** Quantitative analysis of lnc-CRAT40 expression in 103 paired CRC and adjacent normal tissues by qRT-PCR. **D.** Kaplan-Meier survival analysis of overall survival in CRC patients stratified by lnc-CRAT40 expression levels (log-rank test). **E.** Expression of lnc-CRAT40 in unpaired CRC and normal tissues from the TCGA dataset. **F.** lnc-CRAT40 expression levels in normal colon epithelial cells (HCoEpiC) and CRC cell lines. Data are presented as mean ± SD. N, normal tissues; T, tumor tissues. **P<0.01, ***P<0.001, ****P<0.0001.

Total RNA was extracted from tumor tissues and matched adjacent normal tissues obtained from five CRC patients and subjected to RNA sequencing (RNA-Seq). Differential expression analysis (log2Fold Change > 2, *P* < 0.05) identified 420 differentially expressed lncRNAs, including 219 upregulated and 201 downregulated transcripts (Figure 1A, Table S1). The top 10 upregulated lncRNAs were shown in Figure 1B. Among these, LINC02563 was markedly upregulated in gastrointestinal tumors, particularly in CRC, based on The Cancer Genome Atlas (TCGA) dataset analysis (Figure S1A). To validate the RNA-Seq findings, we assessed the expression of LINC02563 in 103 paired CRC and adjacent normal tissues using quantitative reverse transcription polymerase chain reaction (qRT-PCR). Consistent with the RNA-Seq results, LINC02563, also referred to as lnc-CRAT40 (accession number: NR_131984.1), a 277 bp transcript located on chromosome 17q24.1, was significantly elevated in tumor tissues compared with adjacent normal tissues (Figure 1C). Furthermore, coding potential analysis using multiple prediction methods confirmed that lnc-CRAT40 lacks protein-coding ability (Figure S1B).

To explore the clinical relevance of lnc-CRAT40, its expression levels were stratified into high and low groups among the 103 CRC patient samples (Figure S1C). Lnc-CRAT40 expression was found to be significantly associated with lymph node metastasis (*P* = 0.031) (Table 1, Table S2), while no significant correlation was observed with gender, age, tumor location, TNM stages, histological stage, or distant metastasis. Kaplan-Meier survival analysis revealed that high lnc-CRAT40 expression was associated with poor overall survival (*P* = 0.047) (Figure 1D). Additionally, analysis of TCGA data further confirmed that lnc-CRAT40 expression was markedly elevated in CRC tumor tissues compared to normal tissues (Figure 1E), and that high expression levels were significantly associated with distant metastasis (*P* = 0.043) (Table S3). Collectively, these findings suggest that lnc-CRAT40 may serve as a potential biomarker for CRC.

### Lnc-CRAT40 promotes CRC cell proliferation and metastasis in vitro and in vivo

**Figure 2:**
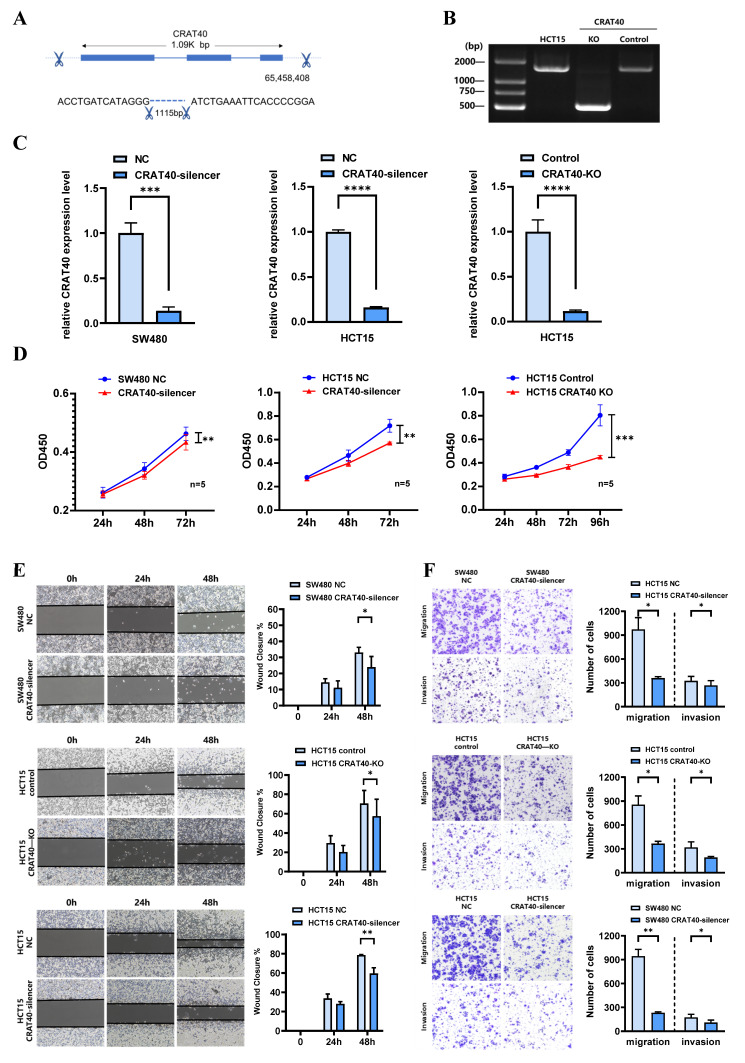
** Silencing of Lnc-CRAT40 inhibits CRC proliferation, migration and invasion in vitro. A.** Schematic of CRISPR/Cas9-mediated knockout of lnc-CRAT40 in HCT15 cells using paired sgRNAs targeting sequences flanking the transcriptional locus, resulting in a 1.1 kb deletion. **B.** Identification of knockout clones by genomic PCR and confirmation of homozygous deletion by Sanger sequencing; off-target regions served as negative controls. **C.** qRT-PCR analysis of lnc-CRAT40 expression in SW480 and HCT15 cells after CRAT40 silencing or control transfection (left), and in control versus CRAT40 knockout HCT15 clones (right). **D.** Cell proliferation assessed by CCK-8 assay following lnc-CRAT40 knockdown or knockout. **E, F.** Wound healing and Transwell assays evaluating migration and invasion in knockdown or knockout cells compared with controls; quantification shown on right. Data represent mean ± SD from three independent experiments. NC, negative control; KO, knockout. *P<0.1, **P<0.01, ***P<0.001, ****p<0.0001.

To explore the potential role of lnc-CRAT40 in CRC, we performed transfection experiments using HCT15 and SW480 CRC cell lines (Figure 1F). Antisense oligonucleotides (ASOs) were employed to knock down lnc-CRAT40 expression, and the CRISPR/Cas9 system was used to generate lnc-CRAT40 knockout (KO) cells (Figure 2A-B). Transfection efficiency was assessed by qRT-PCR (Figure 2C). Our results demonstrated that both knockdown and knockout of lnc-CRAT40 significantly inhibited the proliferation and colony formation abilities of HCT15 cells (Figure 2D, Figure S2A). Additionally, cell cycle analysis revealed G0/G1 phase and/or S phase arrest following knockdown and knockout of lnc-CRAT40 (Figure S2B). Consistent with inhibited proliferation, lnc-CRAT40 silencing also markedly impaired cell migration and invasion, as assessed by wound-healing and transwell assays in HCT15 and SW480 cells (Figures 2E-F). We also performed ectopic overexpression of lnc-CRAT40 in SW480 and HCT15 cells using the pcDNA3.1 expression vector, and confirmed transfection efficiency by qRT-PCR (Figure S2C). Conversely, ectopic overexpression of lnc-CRAT40 in SW480 and HCT15 cells enhanced proliferation, colony formation, and migration abilities (Figures S2C-H), indicating an oncogenic role.

**Figure 3:**
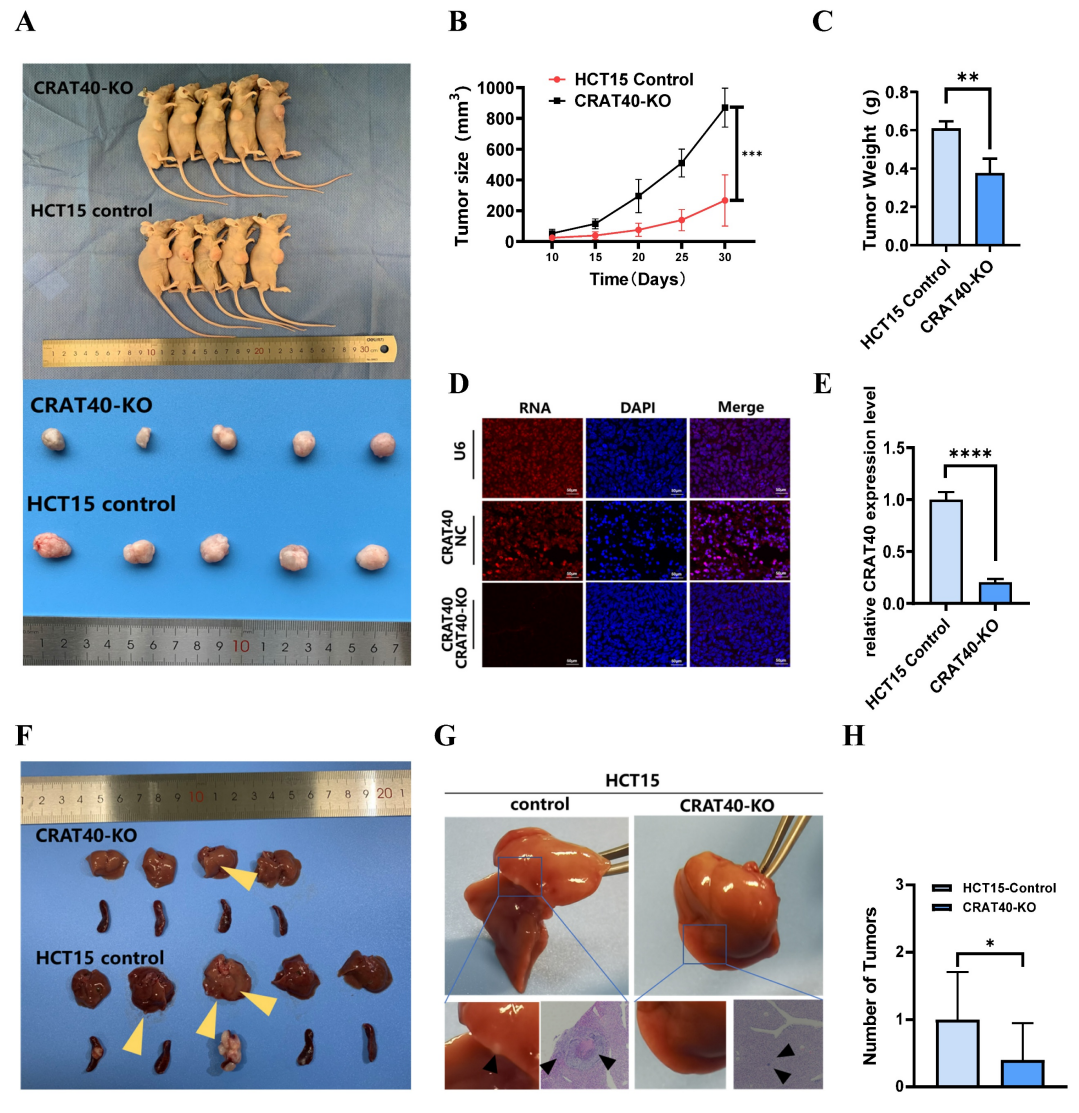
** Knockout of lnc-CRAT40 inhibits CRC proliferation, migration and invasion in vivo. A.** Representative images of xenograft tumors from control and CRAT40 KO groups. **B, C.** Quantification of tumor weights and volumes. Data represent mean ± SD from five independent mice per group. **D.** FISH images showing lnc-CRAT40 signal in tumor tissues from control and KO groups; scale bar, 50 μm. **E.** qRT-PCR detection of lnc-CRAT40 expression in xenograft tumors. **F.** Representative images of liver metastatic nodules following intrasplenic injection. **G.** H&E staining of liver metastatic lesions. **H.** Quantification of metastatic nodules in liver tissue. Data shown as mean ± SD. H&E hematoxylin and eosin; NC, negative control; KO, knockout. *P<0.05, **P<0.01, ***P<0.001, ****P<0.0001.

To investigate the role of lnc-CRAT40 in tumor growth in vivo, we established stable CRAT40 knockout HCT15 cells and generated subcutaneous xenograft models in nude mice. Both the number and size of tumors were significantly reduced in the CRAT40 knockout group compared to the control group (Figure 3A-C). lnc-CRAT40 knockout in tumor tissues was confirmed by qRT-PCR and fluorescence in situ hybridization (FISH) (Figure 3D-E). Confocal microscopy combined with immunofluorescence staining showed a marked reduction in the expression of proliferation markers Ki-67 and PCNA in CRAT40 KO group (Figure S3A-C). In addition, the expression of Cyclin D1 was significantly downregulated in the CRAT40 KO group compared to controls (Figure S3D).

To further explore the effect of lnc-CRAT40 on metastasis in vivo, we established a liver metastasis model by injecting CRC cells into the spleens of nude mice. Remarkably, the CRAT40 KO group exhibited significantly fewer and smaller liver metastatic nodules compared to controls (Figure 3F). Histological examination using hematoxylin and eosin (H&E) staining revealed that the metastatic foci in CRAT40 KO group were notably smaller (Figure 3G-H).

### Lnc-CRAT40 regulates RelA expression in CRC cells

**Figure 4:**
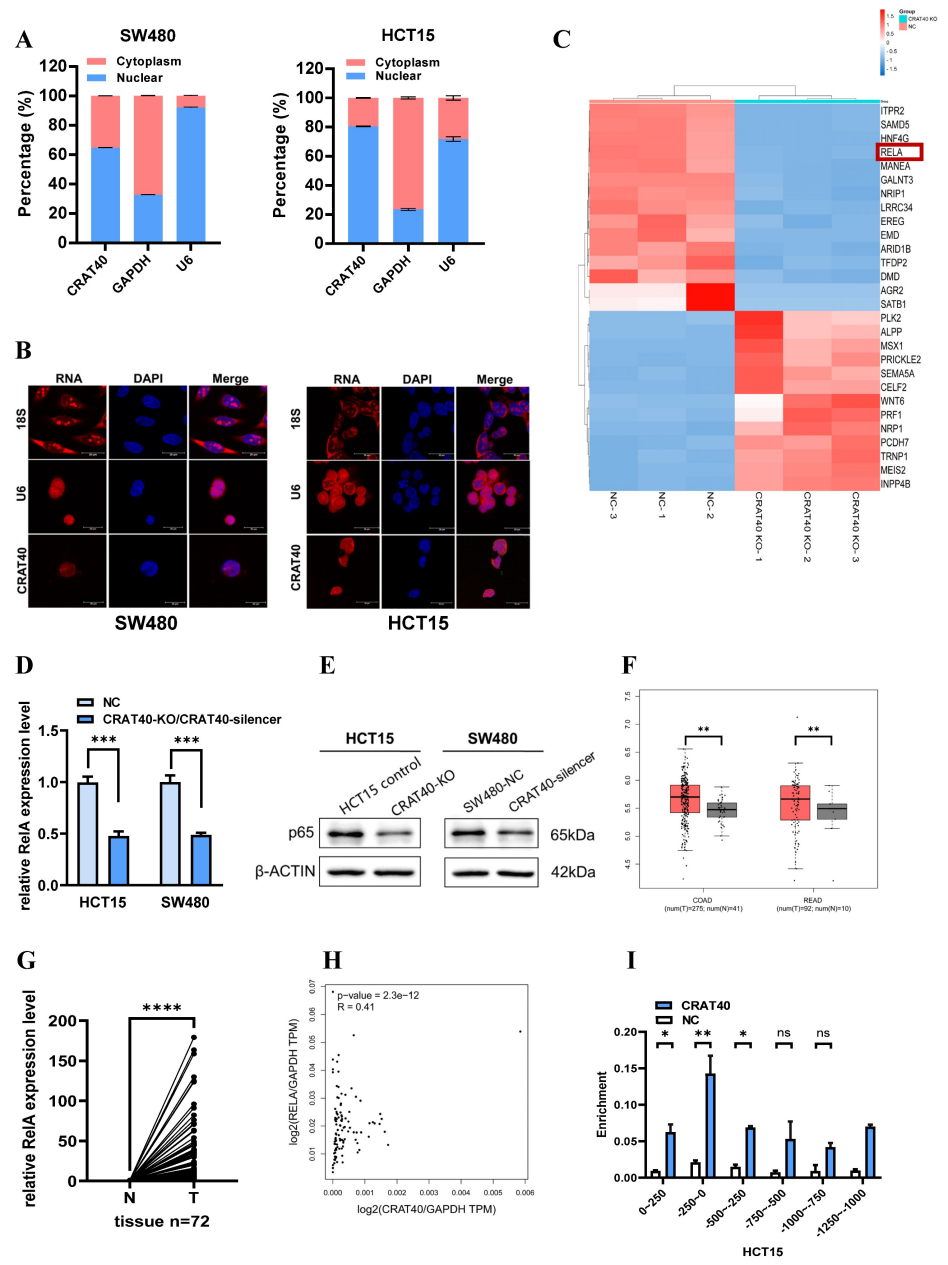
** Lnc-CRAT40 regulates RelA expression in CRC cells. A.** Subcellular fractionation revealing nuclear localization of lnc-CRAT40 in CRC cell lines. **B.** Representative FISH images of lnc-CRAT40 localization in SW480 and HCT15 cells (red); nuclei stained with DAPI (blue). Scale bar, 20 μm. **C.** Heatmap showing top 15 upregulated and downregulated genes after CRAT40 knockout in HCT15 cells. **D, E.** qRT-PCR and Western blot analyses of RelA mRNA and p65 protein levels following lnc-CRAT40 knockdown or knockout. **F.** Upregulation of RelA in TCGA CRC cohort. **G.** qRT-PCR validation of RelA expression in 72 paired CRC tissues and adjacent noncancerous tissues. **H.** Correlation analysis between lnc-CRAT40 and RelA expression in CRC samples (GEPIA 2 database). **I.** ChIRP assay showing enrichment of lnc-CRAT40 at the RelA promoter region in HCT15 cells. COAD, colon adenocarcinoma; READ, rectum adenocarcinoma; N, normal tissues; T, tumor tissues; NC, negative control; ChIRP, chromatin isolation by RNA; ns, not significant. *P<0.05, **P<0.01, ***P<0.001, ****P<0.0001.

Given the importance of subcellular localization in determining lncRNA function, we first employed nucleoplasmic separation and FISH to identify the localization of lnc-CRAT40 in CRC cells. Both assays indicated that lnc-CRAT40 was predominantly localized in the nucleus (Figure 4A-B). This observation led us to hypothesize that lnc-CRAT40 exert its regulatory function through interactions with transcription factors. Sequencing analysis revealed 525 significantly downregulated differentially expressed genes (DEGs) in HCT15 cells following lnc-CRAT40 knockout (Table S4). Gene ontology (GO) enrichment analysis revealed that many of these DEGs were associated with transcription factor activity (Figure S4A). Additionally, Kyoto Encyclopedia of Genes and Genomes (KEGG) pathway further analysis identified several significantly enriched signaling pathways, including transcriptional dysregulation in cancer (Figure S4B). Among the top ten downregulated DEGs, the RelA gene, which encodes the NF-κB p65 subunit known to regulate cancer progression, attracted particular attention (Figure 4C). Western blot and qRT-PCR analysis further confirmed that RelA expression was significantly reduced following lnc-CRAT40 knockout (Figure 4D-E, Table S5).

Subsequent analysis using the TCGA database demonstrated that RelA expression was significantly upregulated in CRC tissues compared to adjacent non-tumor tissues, and its upregulation was correlated with poor prognosis (Figure 4F, Figure S4C). Validation using our own samples further confirmed the elevated expression of RelA in CRC tumor tissues (Figure 4G). Correlation analysis based on TCGA data demonstrated a positive association between lnc-CRAT40 and RelA expression levels (Figure 4H). To explore whether lnc-CRAT40 directly regulates RelA transcription, we performed the chromatin isolation by RNA (ChIRP) assay in HCT15 cells. The results demonstrated that lnc-CRAT40 was enriched at the promoter region of RelA, specifically from -250 bp upstream to the transcription start site (TSS) (Figure 4I), indicating a potential role in RelA transcriptional regulation.

### Lnc-CRAT40 binds specifically to YBX1

**Figure 5:**
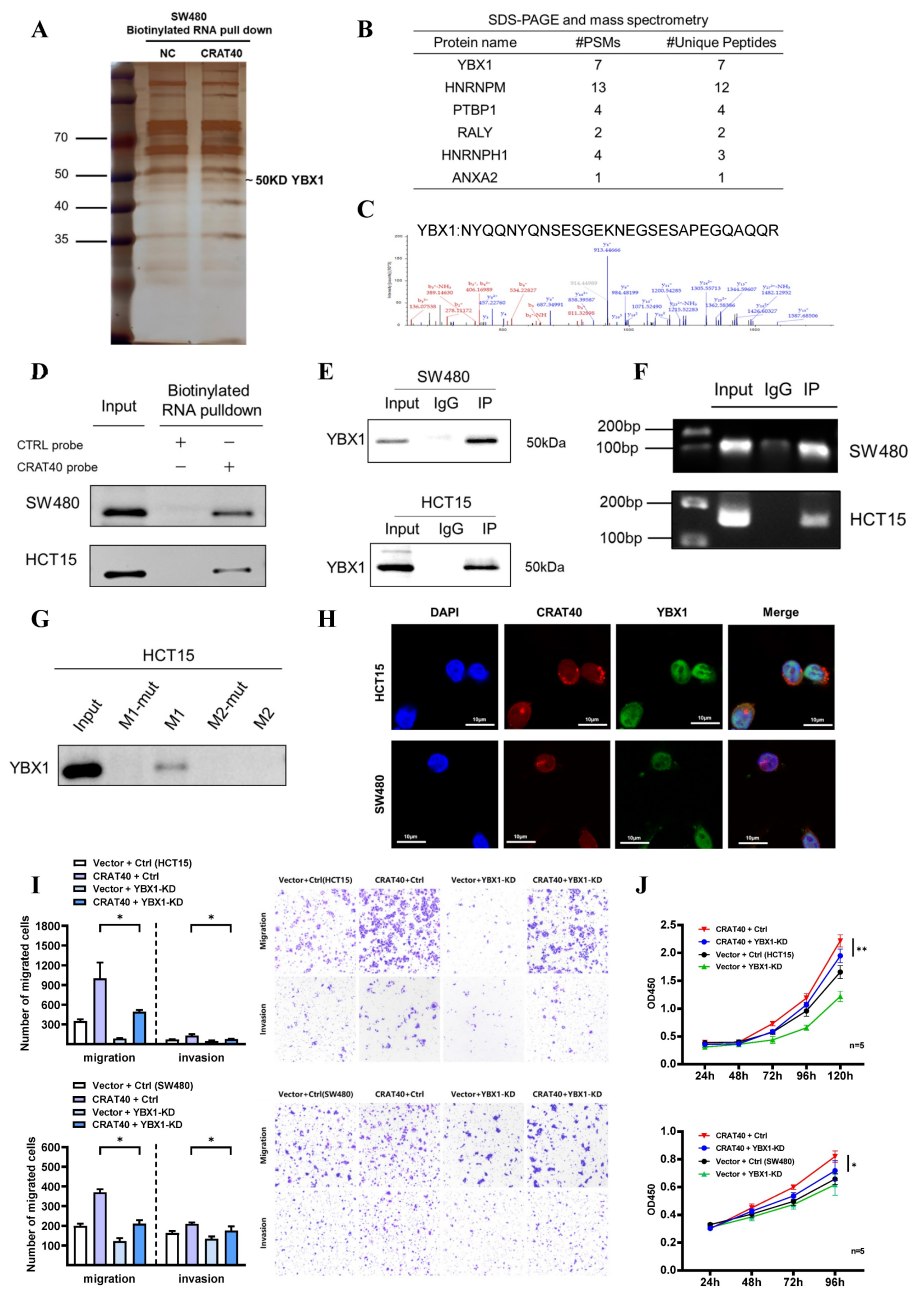
** Lnc-CRAT40 interacts with YBX1. A, B.** Silver staining of proteins pulled down from SW480 cells using biotin-labeled NC or lnc-CRAT40. The arrow indicates the band corresponding to YBX1, identified by mass spectrometry. **C.** Secondary mass spectrogram of YBX1 peptide identified in pull-down. **D.** Western blot confirming specific binding between YBX1 and lnc-CRAT40 in RNA pull-down assays. **E.** Western blot validating enrichment of YBX1 antibody in RIP assays compared with IgG control. **F.** RIP-qPCR showing enrichment of lnc-CRAT40 in YBX1 immunoprecipitates from SW480 and HCT15 cells. **G.** RNA pull-down using wild-type and mutant lnc-CRAT40 probes assessed for YBX1 binding. **H.** Co-localization of lnc-CRAT40 (FISH) and YBX1 (immunofluorescence) in CRC cells. **I, J.** Proliferation, migration, and invasion assays showing that YBX1 knockdown reverses phenotypes induced by lnc-CRAT40 overexpression. Data are presented as means ± SD and were analyzed using Student's t-test; all experiments were performed in triplicate. NC, negative control; KD, knockdown; Ctrl, control; mut, mutant; IP, immunoprecipitation. *P<0.1, **P<0.01.

RNA pulldown using SW480 cell lysates, followed by mass spectrometry, identified multiple protein interactions (Figure 5A-C, Table S6). Among the 22 proteins with specific peptide enrichment, YBX1 emerged as a key candidate due to its high centrality and extensive connections with several other genes, as revealed by protein-protein interaction network analysis (Figure S4D-E). RNA pulldown followed by western blotting confirmed that the biotin-labeled lnc-CRAT40 efficiently precipitated endogenous YBX1 in both SW480 and HCT15 cell lysates (Figure 5D). This interaction was further validated by RNA immunoprecipitation (RIP) assays (Figure 5E-F). Motif analysis suggested that RNA sequences containing the motif 5'-CUGCG-3' may mediate interact with YBX1 (Figure S4E-F). This sequence was identified in two regions of lnc-CRAT40, and subsequent pulldown assays using mutant constructs confirmed that this motif was essential for YBX1 binding (Figure. 5G). These findings suggested a direct and specific interaction between lnc-CRAT40 and YBX1.

Furthermore, we explored the role of YBX1 in CRC. TCGA data revealed significantly elevated YBX1 expression in CRC tissues compared to adjacent normal tissues (Figure S5A). Functional experiments demonstrated that YBX1 silencing significantly inhibited cell proliferation and migration, and induced G0/G1 phase arrest in both SW480 and HCT15 cell lines (Figure S5B-G). Confocal microscopy further showed that lnc-CRAT40 and YBX1 were co-localized in the perinuclear region of CRC cells (Figure 5H). Notably, although lnc-CRAT40 and YBX1 expression were positively correlated in predictive datasets (Figure S5H), knockout of lnc-CRAT40 in HCT15 cells did not significantly affect YBX1 mRNA levels. Collectively, these results suggested that lnc-CRAT40 did not regulate YBX1 transcriptionally, but likely modulated its activity through direct interaction, thereby influencing downstream gene transcription involved in CRC progression. Supporting this, rescue experiments demonstrated that YBX1 knockdown reversed CRAT40 overexpression-induced proliferation and migration in CRC cells (Figure 5I-J), indicating YBX1 is an essential effector of lnc-CRAT40 function.

### Lnc-CRAT40 recruits YBX1 to co-activate the transcription of RelA

**Figure 6:**
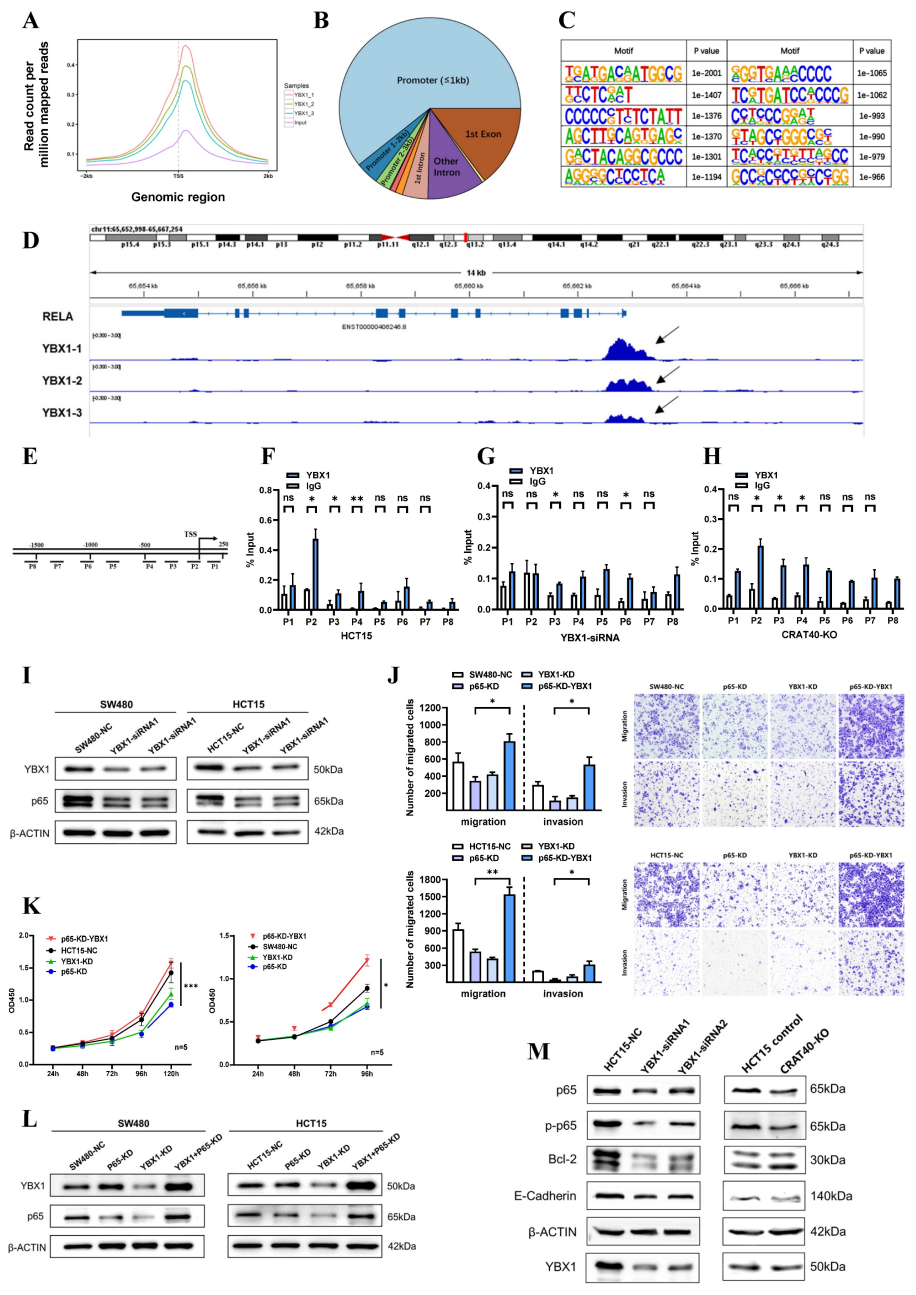
** Lnc-CRAT40 recruits YBX1 to co-activate RelA transcription. A, B.** Genome-wide distribution of YBX1-binding sites in HCT15 cells identified by ChIP-seq, with 60.7% located at promoter regions. **C.** Motif analysis of YBX1 binding sequences. **D.** ChIP-seq tracks showing YBX1 peaks at the RelA promoter. **E.** Schematic of PCR-amplified regions within the RelA promoter for ChIP-qPCR. **F.** ChIP-qPCR confirming specific YBX1 binding to the P2 region of the RelA promoter; IgG as negative control. **G, H.** ChIP-qPCR showing reduced YBX1 enrichment at the RelA promoter after YBX1 knockdown or lnc-CRAT40 knockout. **I.** Western blot showing decreased p65 protein after YBX1 knockdown. **J, K.** Functional rescue assays: RelA knockdown inhibits proliferation, migration, and invasion, which are reversed by YBX1 overexpression. **L.** Western blot analysis of p65 following YBX1 overexpression in RelA-silenced cells. **M.** Western blot of NF/κB and EMT pathway-related proteins, including Bcl-2 and E-cadherin, after YBX1 knockdown and lnc-CRAT40 knockout. ChIP-seq, chromatin immunoprecipitation sequencing; KD, knockdown; NC, negative control; TSS, transcription start site; ns, not significant. *P<0.1, **P<0.01, ***P<0.001.

Previous studies have shown that YBX1 plays a crucial role in transcription regulation. To further investigate whether the CRAT40-YBX1 complex functions through transcriptional regulation, we performed chromatin immunoprecipitation sequencing (CHIP-seq) experiments to identify potential DNA targets for YBX1 (Figure 6A, Table S7). The analysis revealed that YBX1-binding regions were widely distributed across the genome, with 60.7% of these regions located in promoter regions (Figure 6B). Motif analysis of these peaks indicated variability in binding sequence preferences (Figure 6C). Among the putative targets, RelA emerged as a compelling candidate. By overlapping RNA-seq and ChIP-seq data, we identified RelA as a downstream target of both lnc-CRAT40 and YBX1 in HCT15 cells.

Further analysis revealed YBX1-binding peaks approximately 500 bp upstream of the RelA transcription start site (TSS), which led to the design of primers targeting specific regions of the RelA promoter (Figure 6D-E). To determine which site YBX1 bound within the RelA promoter, ChIP-qPCR was performed. The results confirmed that YBX1 was enriched at the region spanning -200 bp to the TSS of the RelA promoter (Figure 6F). Moreover, ChIP-qPCR following the knockdown of YBX1 showed a significant reduction in YBX1 enrichment at this region (Figure 6G). Luciferase reporter assays further supported this regulatory relationship. Luciferase reporter assays showed that YBX1 overexpression enhanced luciferase activity driven by a 2 kb RelA promoter fragment, confirming that YBX1 functioned as a transcriptional activator of RelA (Figure S6A).

As lnc-CRAT40 was also enriched at the same promoter region (Figure 4I), we hypothesized that it facilitated YBX1 recruitment to the RelA promoter. To validate this, we knocked out lnc-CRAT40 in HCT15 cells and observed a similar reduction in YBX1 enrichment at the RelA promoter (Figure 6H), supporting their cooperative role in transcriptional activation.

To assess the functional consequence of this regulatory axis, we investigated whether YBX1 overexpression could rescue the phenotype induced by RelA knockdown. As expected, functionally, YBX1 overexpression rescued proliferation and migration defects caused by RelA silencing (Figure 6I-K). Western blot analysis further showed that YBX1 knockdown led to decreased p65 protein levels in both cell lines (Figure 6L, Figure S6B-C). In addition, YBX1 knockdown resulted in decreased RelA protein levels and reduced expression of NF-κB pathway-related proteins, including phosphorylated p65, Bcl-2, and the downstream EMT-associated molecule E-cadherin, phenotypes similar to those observed in lnc-CRAT40 knockout cells (Figure 6M, Figure S6D-E). Collectively, these results indicate that lnc-CRAT40 acts as a scaffold to recruit YBX1 to the RelA promoter, promoting RelA transcription, activating NF-κB signaling, and driving CRC progression.

### METTL3-mediated m⁶A modification stabilizes lnc-CRAT40 transcript

**Figure 7:**
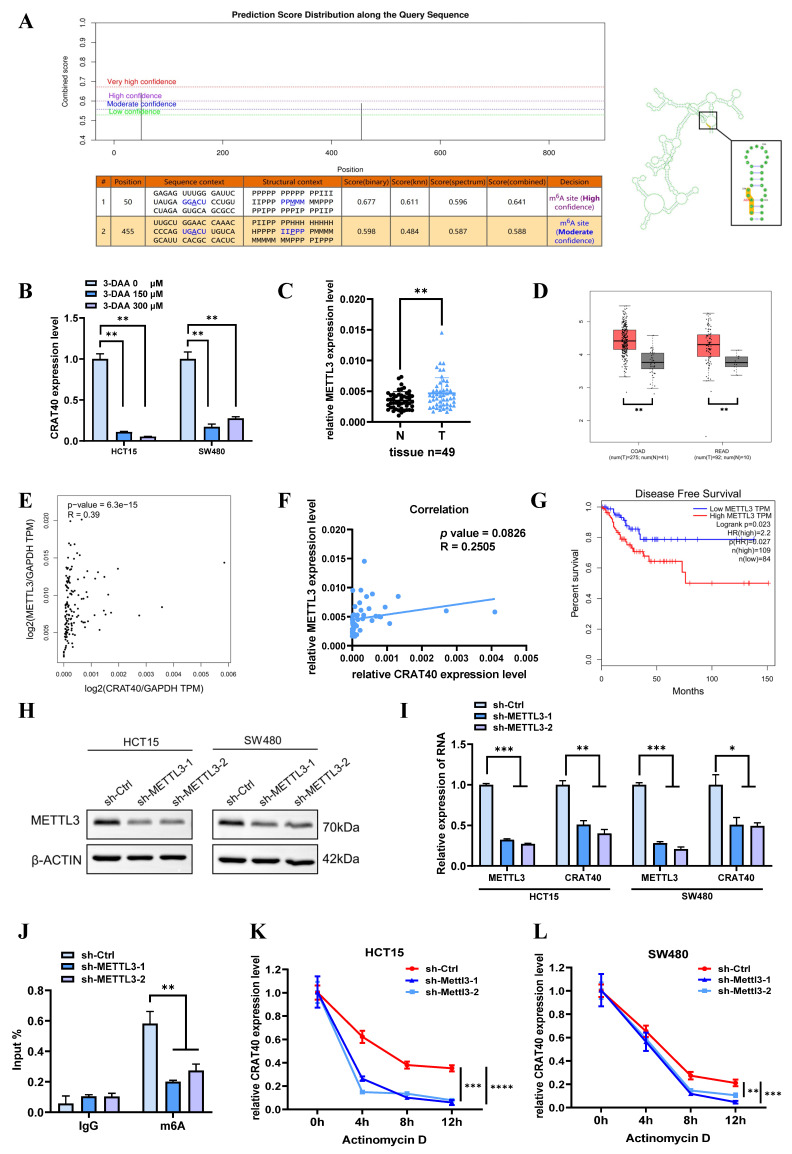
** Lnc-CRAT40 is upregulated in CRC via METTL3-mediated m6A modification. A.** Predicted m6A modification sites within lnc-CRAT40 transcript (SRAMP database). **B.** The decrease of lnc-CRAT40 expression in HCT15 and SW480 cells treated with methylation inhibitor 3-DAA for 24 h (qRT-PCR). **C.** Quantitative analysis of METTL3 expression in 49 paired CRC and adjacent normal tissues by qRT-PCR. **D.** Elevated METTL3 expression in CRC tissues (TCGA dataset). **E.** Correlation between lnc-CRAT40 and METTL3 expression in CRC tissue (n=49). **F.** Positive correlation between lnc-CRAT40 and METTL3 expression in CRC (GEPIA 2). **G.** Kaplan-Meier analysis showing worse disease-free survival in CRC patients with high METTL3 expression. **H.** Western blot confirming METTL3 knockdown efficiency in CRC cells. **I.** qRT-PCR showing reduced METTL3 and lnc-CRAT40 expression upon METTL3 knockdown. **J.** MeRIP-qPCR demonstrating decreased m6A modification on lnc-CRAT40 following METTL3 knockdown. **K, L.** RNA stability assay showing shortened half-life of lnc-CRAT40 transcript after METTL3 knockdown. Data are mean ± SD of three independent experiments. *P < 0.1, **P < 0.01, ***P < 0.001, ****P < 0.0001.

Nevertheless, the underlying mechanism leading to the increased expression of lnc-CRAT40 remains unclear. Initial research has indicated that m6A modifications, extensively present in both mRNA and lncRNA, play a pivotal role. Bioinformatic predictions via the SRAMP website identified multiple m6A sites distributed within the lnc-CRAT40 sequence (Figure 7A). To determine whether m⁶A modification contributed to lnc-CRAT40 upregulation, we treated HCT15 and SW480 cells with the methylation inhibitor 3-deazaadenosine (3-DAA). This treatment significantly reduced lnc-CRAT40 expression levels (Figure 7B), suggesting that m⁶A methylation may be involved in its stability or accumulation.

Given that METTL3 is a key m^6^A methyltransferase, we next examined its expression and potential correlation with lnc-CRAT40 levels using data from 49 paired CRC and adjacent normal tissues, as well as TCGA. METTL3 expression was significantly upregulated in CRC tissues (Figure 7C-D). In the TCGA dataset, METTL3 expression showed a significant positively correlation with lnc-CRAT40 levels (R=0.39, *P*≤0.001, Figure 7E), and a similar trend was observed in our clinical cohort (R = 0.25, *P* = 0.0826, Figure 7F). Additionally, high METTL3 expression was associated with poor disease-free survival, highlighting its prognostic value in CRC (Figure 7G). To further elucidate the regulatory role of METTL3 on lnc-CRAT40, we performed shRNA knockdown of METTL3 in HCT15 and SW480 cells, which led to a apparently decrease in lnc-CRAT40 expression (Figure 7H-I). Methylated RNA immunoprecipitation (MeRIP) assays further confirmed a significant reduction in m^6^A enrichment on lnc-CRAT40 following knockdown of METTL3 in HCT15 cell (Figure 7J). To assess whether m⁶A affected transcript stability, we blocked de novo RNA synthesis with actinomycin D. It revealed that knockdown of METTL3 significantly shortened the half-life of the lnc-CRAT40 transcript (Figure 7K-L), indicating that m⁶A modification contributed to transcript stabilization. Overall, these findings demonstrated that METTL3-driven m^6^A modification stabilizes lnc-CRAT40 in CRC, thereby promoting its upregulation and potentially contributing to CRC progression.

## Discussion

Emerging evidence suggests that lncRNAs play a critical role in the progression of various human malignancies. In our study, we identified lnc-CRAT40 as significantly upregulated in CRC, with higher expression levels correlating with poor prognosis. Functional assays in vitro and in vivo confirmed that lnc-CRAT40 promotes CRC cell proliferation and migration, thereby highlighting its oncogenic potential. Mechanistically, we found that methylation-mediated lnc-CRAT40 acts as a molecular scaffold, recruiting YBX1 to the RELA promoter, subsequently enhancing RELA transcription and activating NF-κB signaling.

The subcellular localization of lncRNAs is often indicative of their mechanistic roles^19^. Predominantly nuclear lncRNAs regulate gene expression through interactions with chromatin, RNA, or protein complexes^20^, ^21^. Our findings that lnc-CRAT40 is enriched in the nucleus align with its function in transcriptional regulation. Supporting this, pathway analyses of lnc-CRAT40 knockout cells revealed dysregulation of transcription factor networks, and RNA pull-down coupled with RNA immunoprecipitation confirmed direct interaction between lnc-CRAT40 and YBX1, a multifunctional RNA-binding protein. These data contribute to the growing understanding that lncRNAs commonly act as scaffolds to recruit transcriptional regulators to specific genomic loci, thereby modulating gene expression in a spatially and temporally precise manner^22^.

Expanding on this paradigm, nuclear lncRNAs such as HOTTIP and A-ROD have been shown to assemble RNA-protein complexes that promote chromatin remodeling and facilitate transcription factor binding, thereby enhancing transcriptional activation^23^,^24^. Furthermore, lncRNAs may contribute to the formation of transcriptional condensates and modulate 3D genome organization^25^. Within this framework, our results demonstrate that lnc-CRAT40 recruits YBX1 to the RELA promoter, facilitating its transcriptional activation. Notably, while YBX1 is known to regulate transcription broadly, its recruitment to RELA by a lncRNA has not been previously reported, revealing a novel regulatory axis in CRC.

YBX1, an RNA-binding protein, plays critical roles in various cellular functions, including transcriptional regulation, DNA repair, RNA splicing, mRNA translation, and stability^26^-^28^. Previous studies have shown that YBX1 promotes tumor progression in breast, bladder, and hematological cancers and is linked to poor prognosis^29^-^31^. Increasing evidence indicates that lncRNAs interact with YBX1 in the pathophysiology of CRC. These mechanisms include enhancing YBX1 protein stability, regulating its subcellular localization, and recruiting it to specific gene promoters to drive transcriptional activation. For example, MNX1-AS1 binds to YBX1 and prevents its ubiquitination-mediated degradation^32^. Similarly, EVADR acts as a molecular scaffold to protect YBX1^33^. Beyond stabilization, certain lncRNAs facilitate the targeted recruitment of YBX1 to specific gene loci. POU6F2-AS1 recruits YBX1 to the *FASN* promoter^10^. Likewise, RP11-296E3.2 interacts with YBX1 and directs it to the STAT3 promoter, leading to increased STAT3 transcription and phosphorylation^33^. Collectively, these studies highlight the diverse mechanisms by which lncRNAs collaborate with YBX1 to regulate cancer progression, warranting further investigation into these potential pathways. In our study, we show that lnc-CRAT40 functions similarly by recruiting YBX1 to the RELA promoter, enhancing NF-κB signaling and facilitating CRC progression.

The NF-κB family of inducible transcription factors regulates key biological processes, including immune and inflammatory responses, cell differentiation, and proliferation^35^, ^36^. RelA (p65), a central subunit of the NF-κB pathway, plays a crucial role in controlling tumor progression in various cancers, including CRC^37^-^39^. RelA promotes tumor growth, anti-apoptotic responses, and invasion by activating NF-κB signaling and regulating the expression of genes associated with cell proliferation and survival, such as Cyclin D1 and Bcl-2^40^, ^41^. Although numerous studies have provided indirect evidence of a functional collaboration between YBX1 and RelA, our study offers the first direct evidence that YBX1 regulates RelA transcription, playing a pivotal role in CRC progression. Additionally, other lncRNAs such as HOTAIR have been shown to promote NF-κB activity by facilitating p65 nuclear translocation, highlighting multiple layers by which lncRNAs modulate this critical pathway^42^.

Epitranscriptomic regulation, particularly m⁶A modification, has recently emerged as a pivotal mechanism controlling RNA metabolism and function^43^, ^44^. However, m⁶A-mediated regulation of lncRNAs in CRC remains underexplored. Our study reveals that METTL3-dependent m⁶A methylation stabilizes lnc-CRAT40 transcripts, contributing to its upregulation and oncogenic function. This finding aligns with growing evidence that m⁶A writers, readers, and erasers dynamically modulate cancer-associated lncRNAs, affecting their stability, localization, and interactions. Beyond m⁶A, other epigenetic mechanisms such as DNA methylation and histone modifications may also regulate lnc-CRAT40 expression, warranting future investigation.

Several limitations in our study should be acknowledged. First, although we identified a specific RNA-protein interaction between lnc-CRAT40 and YBX1, it remains unclear whether lnc-CRAT40 facilitates transcription solely through this interaction or whether it also participates in broader chromatin architectural changes. Whether lnc-CRAT40 exerts similar effects in cis or trans remains an open question and warrants further investigation using chromatin conformation capture techniques such as Hi-C or 4C-seq. Second, whether the regulatory function depends on the mature RNA transcript or is mediated through the act of transcription itself remains unresolved. Future studies should utilize transcript-specific degradation, premature termination, and promoter deletion to dissect these mechanisms. Third, the upstream regulatory mechanisms controlling lnc-CRAT40 expression remain incompletely characterized. Additionally, more direct and quantitative methods—such as ELISA-based detection—would further strengthen the mechanistic foundation. Finally, the clinical relevance and potential as a therapeutic target of the CRAT40-YBX1-RELA axis should be validated in larger, independent cohorts and across diverse CRC subtypes.

In conclusion, we demonstrate that m⁶A-modified lnc-CRAT40 promotes CRC proliferation and metastasis by recruiting YBX1 to activate RelA transcription and NF-κB signaling. METTL3-mediated m⁶A modification plays a crucial role in stabilizing lnc-CRAT40. Our findings not only deepen the understanding of lncRNA-mediated transcriptional regulation in CRC but also highlight the CRAT40-YBX1-RelA axis as a potential target for novel anti-metastatic therapies.

## Supplementary Material

Supplementary figures and tables 2, 3, and 5.

Supplementary table 1.

Supplementary table 4.

Supplementary table 6.

Supplementary table 7.

## Figures and Tables

**Figure 8:**
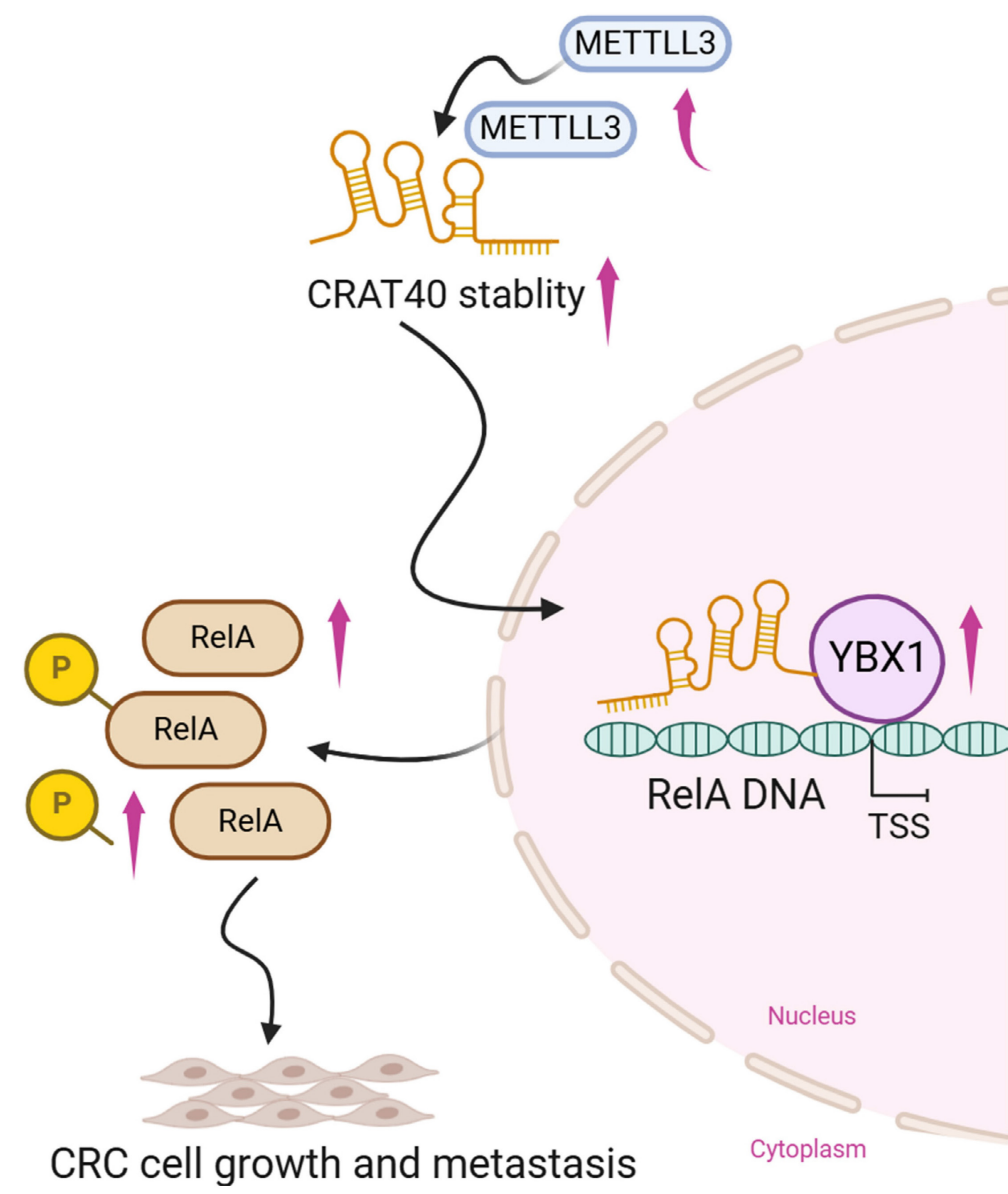
** Schematic model illustrating the mechanism of lnc-CRAT40 in CRC progression.** Schematic overview depicting that METTL3-mediated m6A modification stabilizes lnc-CRAT40, which functions as a scaffold recruiting YBX1 to the RelA promoter, activating NF-κB signaling and promoting colorectal cancer proliferation and metastasis.

**Table 1:** Association between lnc-CRAT40 expression levels and clinicopathological features in colorectal cancer patients (n = 103).

	N (%)	CRAT40 expression		p-value
		high	low	
Gender				0.500
Female	70 (68.0%)	41 (58.6%)	29 (41.4%)	
Male	33 (32.0%)	17 (51.5%)	16 (48.5%)	
Age (years)				0.104
<60	49 (47.6%)	23 (46.9%)	26 (53.1%)	
≥60	54 (52.4%)	35 (64.8%)	19 (35.2%)	
Location				0.105
Colon	39 (37.9%)	18 (46.2%)	21 (53.8%)	
Rectum	64 (62.1%)	40 (62.5%)	24 (37.5%)	
T stage				0.877
T1-T2	19 (18.4%)	11 (57.9%)	8 (42.1%)	
T3-T4	84 (81.6%)	47 (56.0%)	37 (44.0%)	
N stage				0.604
N0-N1	92 (89.3%)	51 (55.4%)	41 (44.6%)	
N2-N3	11 (10.7%)	7 (63.6%)	4 (36.4%)	
M stage				0.801
M0	88 (85.4%)	50 (56.8%)	38 (43.2%)	
M1	15 (14.6%)	8 (53.3%)	7 (46.7%)	
Stage				0.287
I/II	58 (56.3%)	30 (51.7%)	28 (48.3%)	
III/IV	45 (43.7%)	28 (62.2%)	17 (37.8%)	
Lymphatic				0.031
No	61 (59.2%)	29 (47.5%)	32 (52.5%)	
Yes	42 (40.8%)	29 (69.0%)	13 (31.0%)	
Distant metastasis				0.165
No	91 (88.3%)	49 (53.8%)	42 (46.2%)	
Yes	12 (11.7%)	9 (75.0%)	3 (25.0%)	
