# Supplementary material for: Methylation-mediated LncRNA CRAT40 promotes colorectal cancer progression by recruiting YBX1 to initiate RelA transcription
Source: Int J Biol Sci. 2025 Jul 25;21(11):4834–50. doi: 10.7150/ijbs.105629 (PMC12374820; doi:10.7150/ijbs.105629)
Supplement: Supplementary file 1 — Supplementary figures and tables 2, 3, and 5. [file ijbsv21p4834s1.pdf]

SUPPLEMENTARY FIGURE LEGENDS

Figure S1. LncRNA CRAT40 is upregulated in CRC and lacked protein-coding capacity.

A. Expression levels of lnc-CRAT40 across multiple tumor types analyzed using the GEPIA database. Dot plot shows CRAT40 transcript levels (TPM) in tumor and matched normal tissues.

B. Protein-coding potential of CRAT40 predicted by CPAT and CPC2 online tools.

C. CRC samples were divided into high- (n=58) and low- (n=45) expression groups based on median CRAT40 expression.

CPAT, Coding-Potential Assessment Tool; CPC2, Coding Potential Calculator 2.

Figure S2. Lnc-CRAT40 regulates colony formation ability and altered cell cycle progression in CRC cells.

A. Colony formation assays assessing proliferative capacity of HCT15 CRAT40-KO cells and HCT15/SW480 cells transfected with CRAT40 silencers. Quantification shown below.

B. Flow cytometry analysis showing alterations in G0/G1 and S phases after CRAT40 knockdown or knockout (left), with statistical graphs (right).

C. Efficiency of CRAT40 overexpression validated by qRT-PCR.

D, E. Wound healing and Transwell assays in SW480 and HCT15 cells with CRAT40 overexpression versus NC (left); quantification presented (right).

F. Flow cytometry showing cell cycle distribution changes upon CRAT40 overexpression (left), with statistical graphs (right).

G. Colony formation assays after CRAT40 overexpression with quantification.

H. CCK-8 assay measuring proliferation in SW480 and HCT15 cells with or without CRAT40 overexpression.

Data are mean ± SD from three independent experiments. NC, negative control; KO, knockout; ns, not significant. *$P < 0.05$, **$P < 0.01$, ****$P < 0.0001$.

Figure S3. Knockout of Lnc-CRAT40 reduces proliferation marker expression in CRC xenograft tumors.

A. Representative H&E staining of xenograft tumors. Scale bar, 50 μm.

B. IHC staining for PCNA in tumor tissues from control and KO groups with quantification. Scale bar, 50 μm.

C. Immunofluorescence images showing Ki-67 (green) and DAPI (blue) staining in tumor sections. Scale bar, 20 μm.

D. IHC analysis and quantification of Cyclin D1 expression in tumor tissues. Scale bar, 50 μm.

H&E hematoxylin and eosin; IHC, immunohistochemistry; KO, knockout. *$P<0.05$, **$P<0.01$.

Figure S4. Lnc-CRAT40 may be a critical modulator in CRC progress.

A. GO analysis of these DEGs revealed the top 10 DEGs significantly enriched in three different biological processes.

B. KEGG pathway enrichment analysis of DEGs following CRAT40 knockout.

C. Kaplan–Meier analysis correlating RelA expression with overall survival in TCGA CRC cohort.

D. Protein profiling identifying 36 candidate proteins specifically binding CRAT40 by RNA pull-down.

E. Protein–protein interaction network of CRAT40-interacting proteins.

F. Predicted YBX1 RNA-binding motifs from motif analysis.

G. Predicted YBX1 RNA-binding sites from the RBPDB database.

DEGs, differentially expressed genes; GO, Gene Ontology; KEGG, Kyoto Encyclopedia of Genes and Genomes; TCGA, The Cancer Genome Atlas; RBPDB, RNA-binding protein prediction database.

Figure S5. YBX1 silencing inhibits CRC proliferation, migration and invasion in vitro .

A. Upregulation of YBX1 in TCGA CRC cohort.

B. Efficient YBX1 knockdown in SW480 and HCT15 cells validated by qRT-PCR.

C. YBX1 overexpression by lentiviral transduction confirmed by qRT-PCR and Western blot.

D. CCK-8 assay showing reduced proliferation upon YBX1 knockdown.

E, F. Transwell migration and wound healing assays demonstrating impaired migration and invasion after YBX1 silencing; quantification shown.

G. Flow cytometry showing cell cycle alterations after YBX1 knockdown with corresponding statistics.

H. Correlation analysis of CRAT40 and YBX1 expression in CRC (GEPIA 2 website).

Data represent mean ± SD from three independent experiments. NC, negative control; ns, not significant. $^*P<0.05$, $^{**}P<0.01$, $^{***}P<0.001$, $^{****}P<0.0001$.

Figure S6. Statistical analysis of dual-luciferase reporter assays and Western blot data.

A. Luciferase reporter assay showing increased activity of RelA promoter (2 kb fragment) upon YBX1 overexpression in HEK293T cells.

B, C. Western blot analysis of protein expression changes following YBX1 overexpression in p65-knockdown SW480 (B) and HCT15 (C) cells, with quantification based on the relative gray values of the internal reference.

D, E. Western blots showing protein levels of p65, phospho-p65, YBX1, E-Cadherin, and Bcl-2 after YBX1 knockdown (D) and CRAT40 knockout (E). Quantification of gray values normalized to the internal reference. Data are mean ± SD from three independent experiments.

Ctrl, control; NC, negative control; KD, kncokdown; KO, knockout. *P < 0.05, **P < 0.01, ****P < 0.0001.

Figure S1. LncRNA CRAT40 is upregulated in CRC and lacked protein-coding capacity.

A

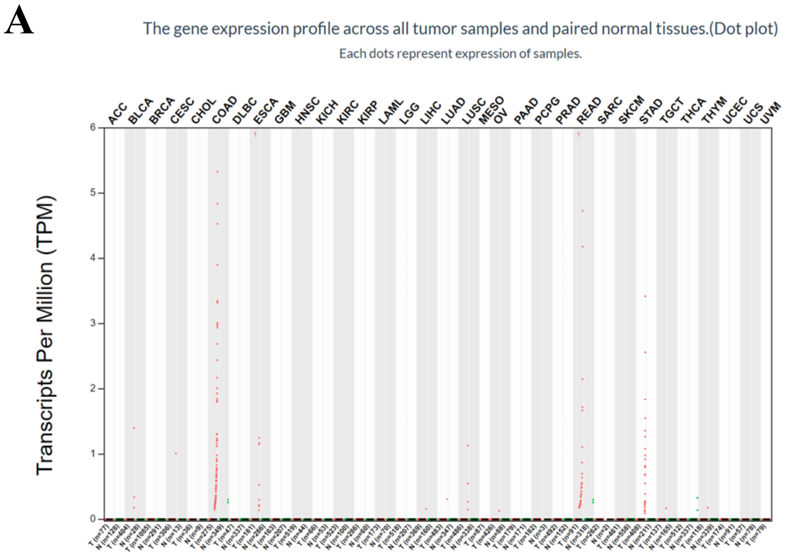

C

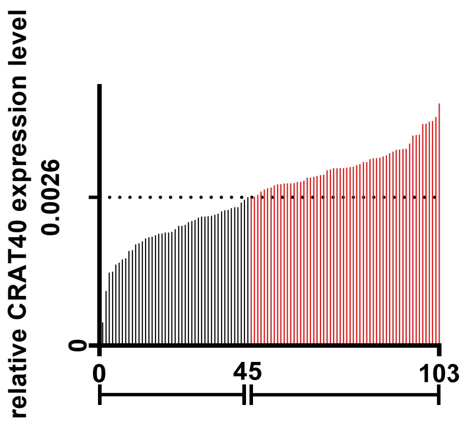

B

ID	Label	Coding probability
ENST00000579474.1	noncoding	0.0615623

Result for species name : hg19 with job ID :1676674025							
Data ID	Sequence Name	RNA Size	ORF Size	Ficket Score	Hexamer Score	Coding Probability	Coding Label
0	ENST00000579474.1	277	87	1.0459	0.043682592378	0.026635381816247	no

Figure S2. Lnc-CRAT40 regulates colony formation ability and altered cell cycle progression in CRC cells.

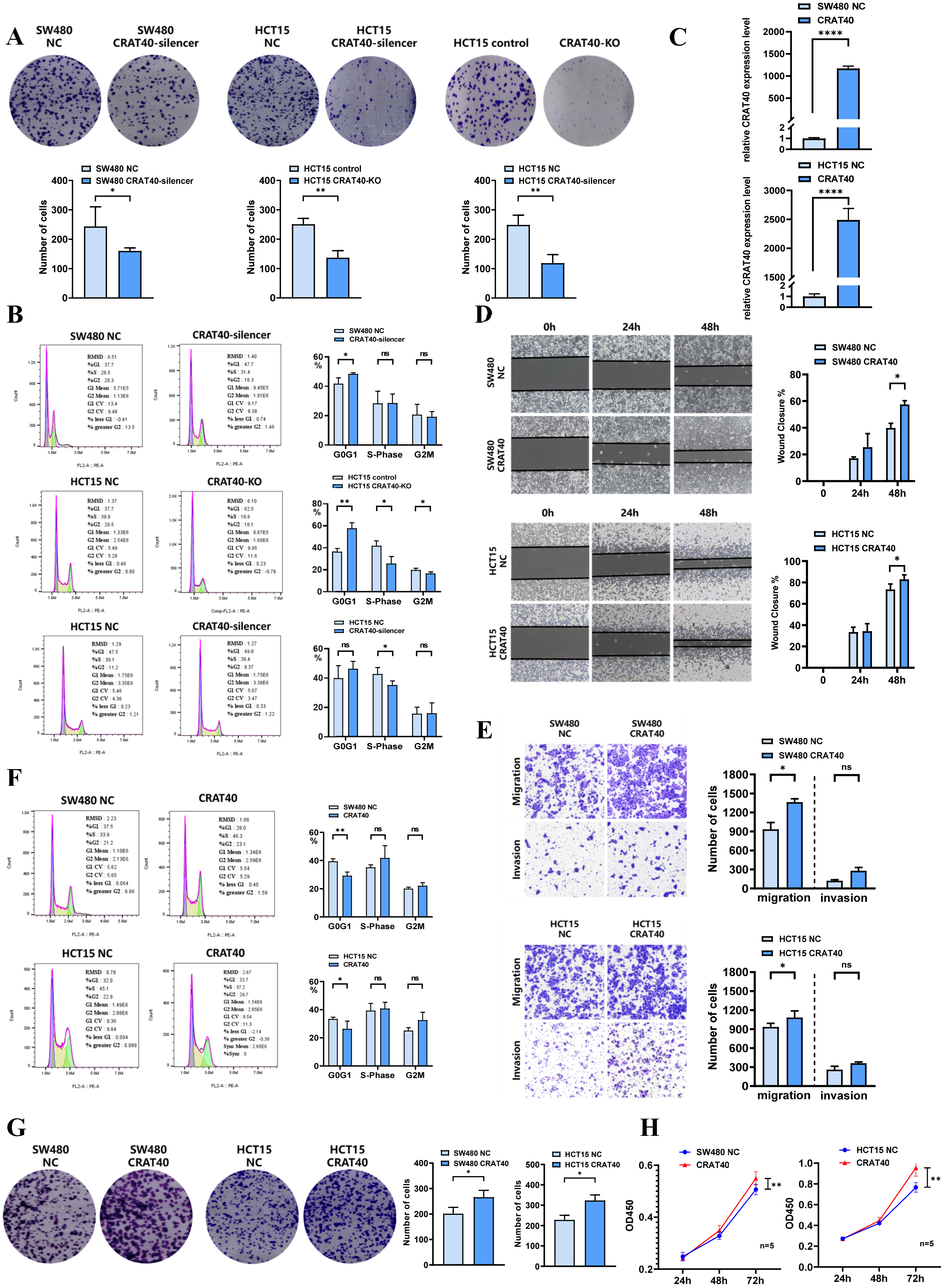

Figure S3. Knockout of Lnc-CRAT40 reduces proliferation marker expression in CRC xenograft tumors.

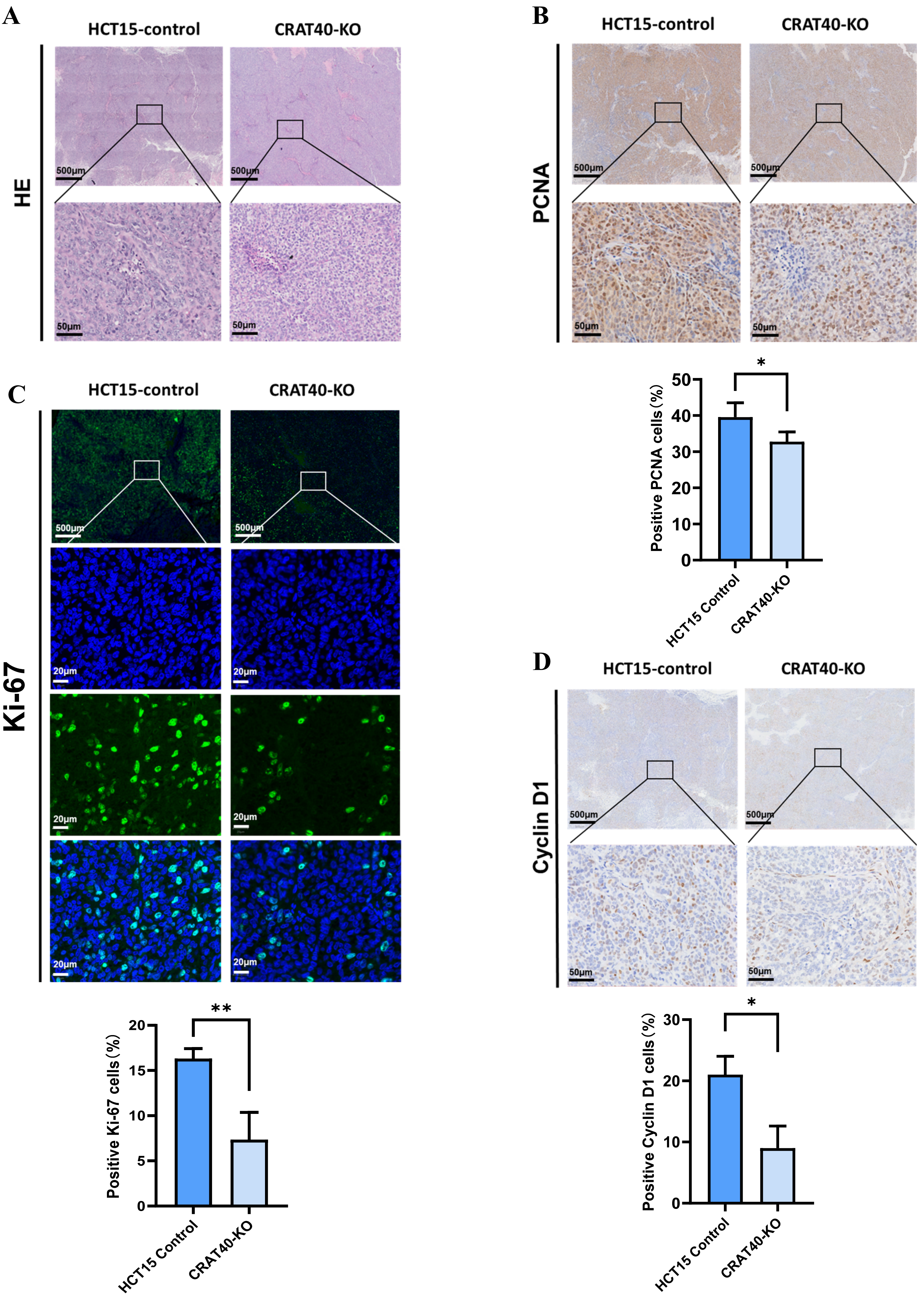

Figure S4. Lnc-CRAT40 may be a critical modulator in CRC progress.

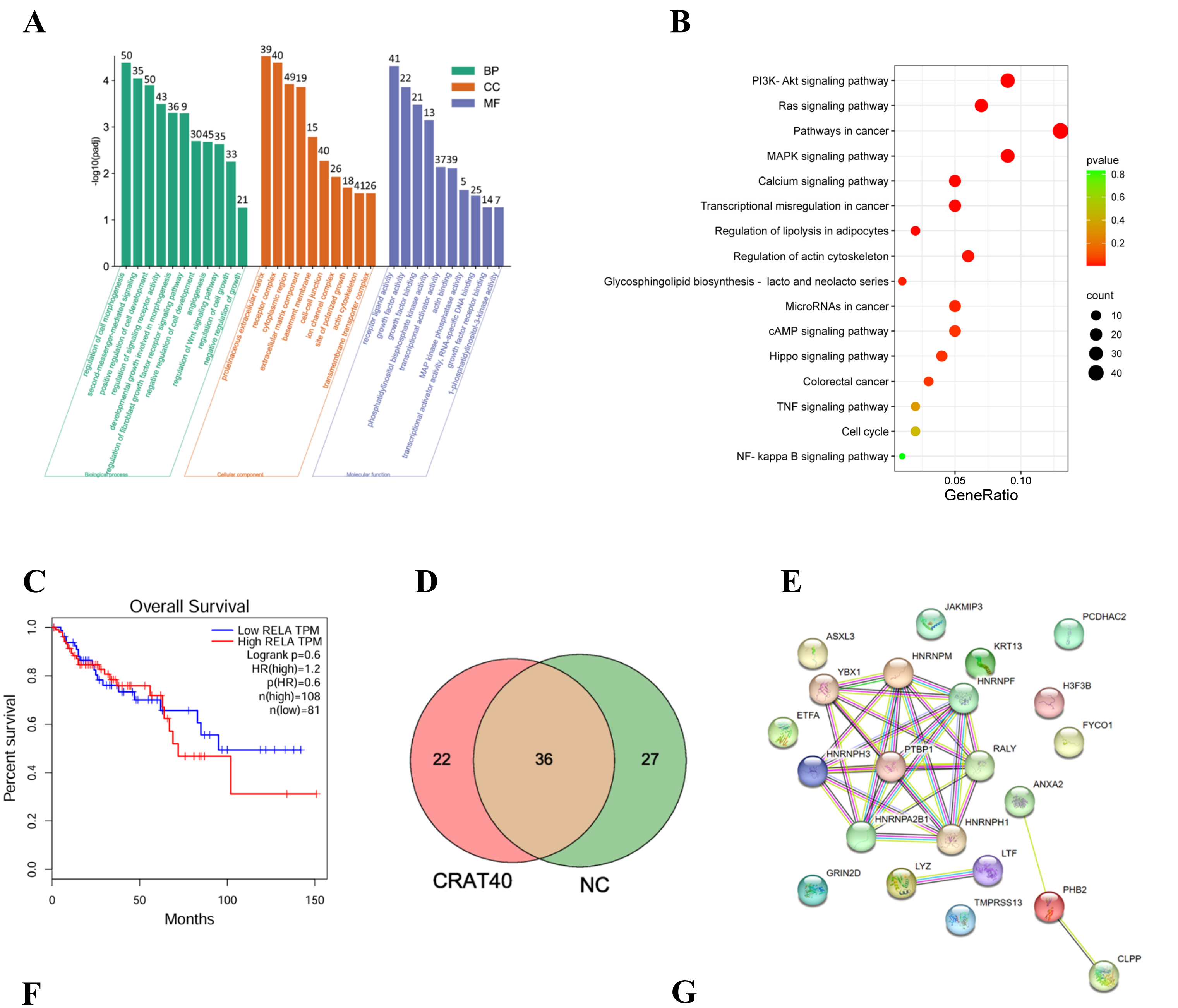

F

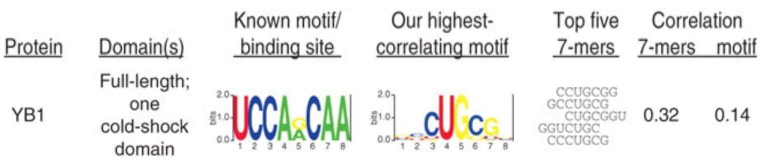

G

Score	Relative Score	RBP Name	Start	End	Matching sequence
6.33890	100%	YBX1	150	155	CCUGCG
5.09233	80%	YBX1	73	78	ACUGCG

Figure S5. YBX1 silencing inhibits CRC proliferation, migration and invasion in vitro.

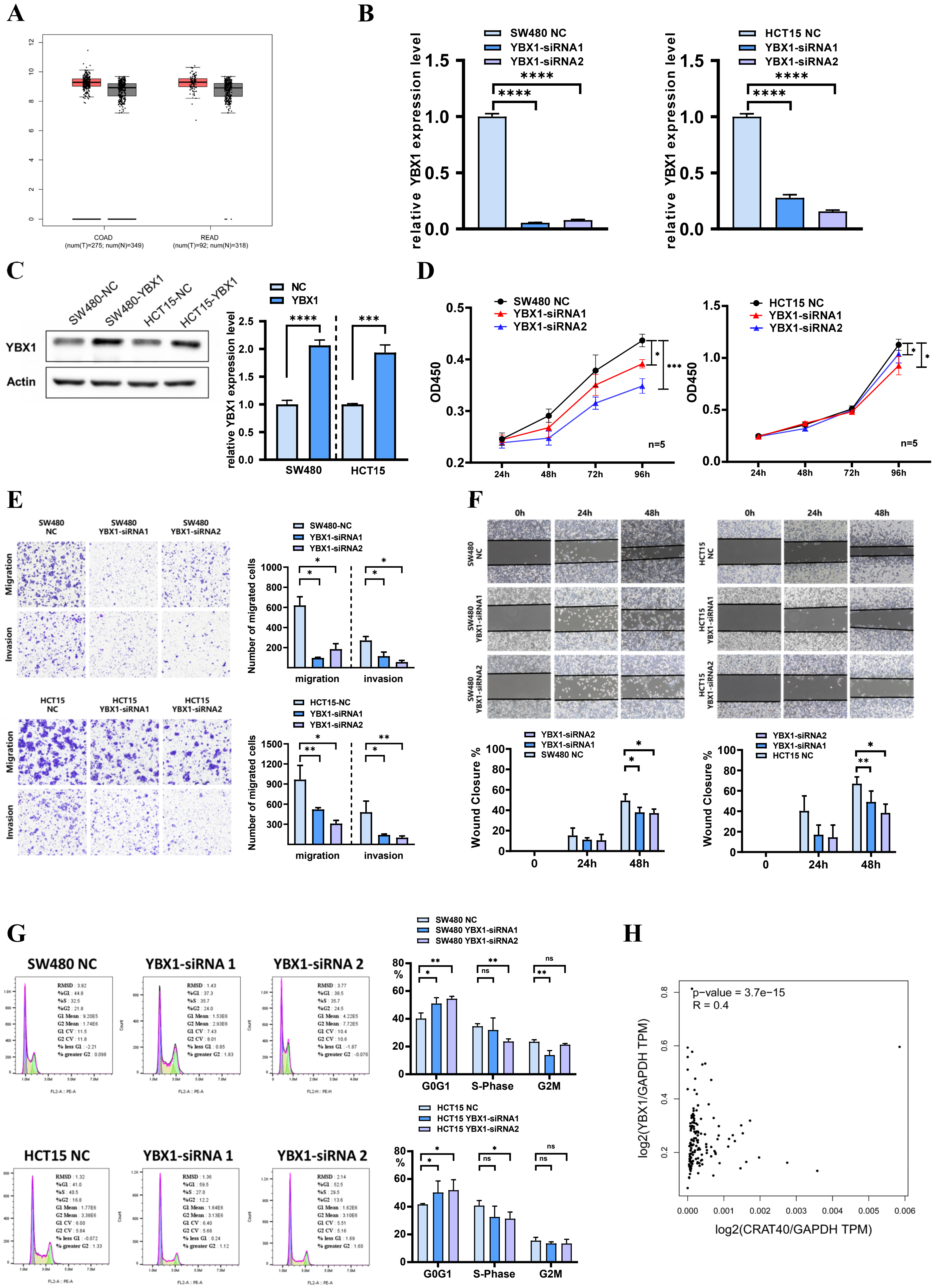

Figure S6. Statistical analysis of dual-luciferase reporter assays and Western blot data.

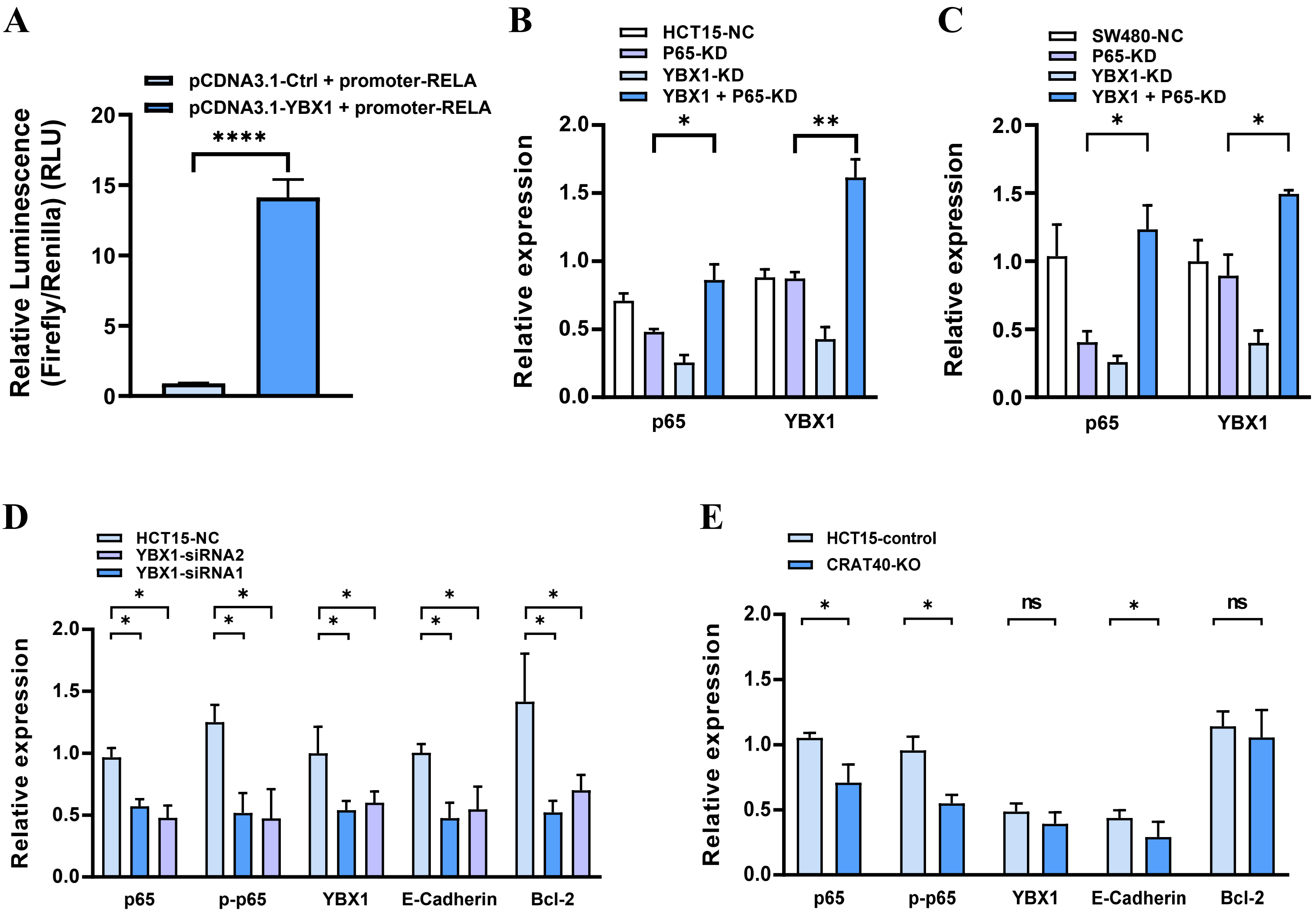

TABLE LEGENDS

Table S1. List of all significantly upregulated and downregulated lncRNAs identified in CRC tissues.

Table S2. Primer sequences used in this study.

Table S3. Clinicopathological characteristics of CRC patients stratified by lnc-CRAT40 expression levels in the TCGA cohort (n = 597).

Table S4. Differentially expressed genes identified by RNA-seq in lnc-CRAT40 knockout HCT15 cells.

Table S5. Antibodies used in this study.

Table S6. Proteins identified by biotinylated oligonucleotide RNA pull-down followed by mass spectrometry in HCT15 cell lysates.

Table S7. Genome-wide distribution of YBX1-binding peaks in HCT15 cells identified by ChIP-seq.

Table S2. All primer sequences.

Primers for qRT-PCR/ PCR detection

Primer	Sequence
CRAT40-F1	Forward: 5’-CTGCGAGGGTGGTCCTTTAT-3’
CRAT40-R1	Reverse: 5’-TCTTGAATGGAGCTGGGCAC-3’
CRAT40-F2	Forward: 5’-GTGGAAACTGAGCGTCCTCT-3’
CRAT40-R2	Reverse: 5’-CGTTAATTGCGCCTCTCATCC-3’
CRAT40-R3	Forward: 5’-TAGGTGCTCAGGGCCTAATG-3’
CRAT40-F3	Reverse: 5’-AGGAGGCGGTGTAGGTTCTT-3’
U6-F	Forward:5’-CGCTTCGGCAGCACATATAC-3’
U6-R	Reverse: 5’-AGGGGCCATGCTAATCTTCT-3’
β-Actin-F	Forward: 5’-CTTCGCGGGCGACGAT-3’
β-Actin-R	Reverse: 5’-ATAGGAATCCTTCTGACCCATGC-3’
YBX1-R	Forward: 5’-CGAAGGTACTTCCTGGGGTTA-3’
YBX1-F	Reverse: 5’-GGGGACAAGAAGGTCATCGC-3’
YBX1-F1	Forward: 5’-GGACAAGAAGGTCATCGCAAC-3’
YBX1-R1	Reverse: 5’-TCTCCATCTCCTACACTGCGA-3’
Primer-YBX1-PCR-F	Forward: 5’-GGATCTATTTCCGGTGAATTCATGAGCAGCGA GGCCGAG-3’
Primer-YBX1-PCR-R	Reverse: 5’-GGAGGGAGAGGGGCGGGATCCTTACTCAGCC CCGCCCTGCTCAGCCTCGGG-3’

Primers for qRT-PCR after CHIP/CHIRP.

Primer	Sequence
0 ~ 250 bp	Forward: GCCATGGACGGTGAGGTC
0 ~ 250 bp	Reverse: GCAGGAAGGGGCGGAAAG
-250 ~ 0 bp	Forward: CGGCTGTGCGTGCAG
-250 ~ 0 bp	Reverse: ATTCGCCAGAGGCGGAAATG
-500 ~ -250 bp	Forward: CTTTAGCGGTTAGGAAACCCG

-500 ~ -250 bp	Reverse: TTGGTGATGTCACTCTGGGC
-750 ~ -500 bp	Forward: AGGCTTCAGAGACGAGGCT
-750 ~ -500 bp	Reverse: TCCCAGCCTGACAGTGCATCAA
-1000 ~ -750 bp	Forward: GAGCCTGGGAAACAAGTCAGAA
-1000 ~ -750 bp	Reverse: GGCTGACCACTAATTCTGTCATG
-1250 ~ -1000 bp	Forward: TCCATTCTGTCACCTGAAGTCG
-1250 ~ -1000 bp	Reverse: ACTTGTTTCCCAGGCTCTGAAG
P8-F	Forward: CCTTGAGTTTTGCAATGGGTAC
P8-R	Reverse: GCCGACTTCAGGTGACAGAAT
P7-F	Forward: GAGCCTGGGAAACAAGTCAGAA
P7-R	Reverse: AGGCCTCATCCCCAAAGGA
P6-F	Forward: CATGACAGAATTAGTGGTCAGCC
P6-R	Reverse: AGCCTCGTCTCTGAAGCCT
P5-F	Forward: ATCGTAAGCTGGCGCTTTAGCT
P5-R	Reverse: TCCCAGCCTGACAGTGCATCAA
P4-F	Forward: AGATTGGGGTGGGTAGGACA
P4-R	Reverse: CGGGTTTCCTAACCGCTAAAG
P3-F	Forward: TGACATCACCAAACTCCGCCGA
P3-R	Reverse: GTGGGTCCGCCGATTACTCA
P2-F	Forward: CGGCTGTGCGTGCAG
P2-R	Reverse: ATTCGCCAGAGGCGGAAATG
P1-F	Forward: GGTGAGGTCGCCCTCTGAC
P1-R	Reverse: GCAGGAAGGGGCGGAAAG

Sequence of siRNAs/shRNAs.

Primer	Sequence
CRAT40-siRNA1	5’-GTGGTCCTTTATTGTCAGA-3’
CRAT40-siRNA2	5’-GACATGCAGTGGAAACTGA-3’
CRAT40-siRNA3	5’-GAGGCGCAATTAACGTTAT-3’

CRAT40-ASO1	5'-ATGAGGACTCCTGTCTAGAG-3'
CRAT40-ASO2	5'-CCATTCAAGAAAGCACACCT-3'
CRAT40-ASO3	5'-AAGGAAAACCCAGTGGCCGT-3'
YBX1-siRNA1	5'-GGACGGCAATGAAGAAGAT-3'
YBX1-siRNA2	5'-CCACGCAATTACCAGCAAA-3'
YBX1-siRNA3	5'-GCAGACCGTAACCATTATA-3'
METTL3-shRNA-F1	5'-CCGGAAGTATGTTCACTATGAAACTCGAGTTT CATAGTGAACATACTTTTTTT-3'
METTL3-shRNA-R1	5'-AATTAAAAAAAGTATGTTCACTATGAAACTC GAGTTTCATAGTGAACATACTT-3'
METTL3-shRNA-F2	5'-CCGGCAAGGAACAATCCATTGTTCTCGAGAA CAATGGATTGTTCCTTGTTTTT-3'
METTL3-shRNA-R2	5'-AATTAAAAACAAGGAACAATCCATTGTTCTCG AGAACAATGGATTGTTCCTTG-3'
RelA-siRNA1	5'-GCCTTAATAGTAGGGTAAGTT-3'
RelA-siRNA2	5'-CGGATTGAGGAGAAACGTAAA-3'

Sequence of sgRNAs.

Primer	Sequence
CRAT40-gRNA-A1	Forward: 5'-AAGCGGCTCACCAGGACCGA-3'
CRAT40-gRNA-A2	Forward: 5'-TGGGGAGAACTGGACCCTAT-3'

Sequence of RNA pull down/RIP.

Primer	Sequence
CRAT40-RPL1	5'-TGCTTTCTTGAATGGAGCTGGGCAC-3'Biotin
CRAT40-RPL-NC	5'-ACGAAAGAACTTACCTCGACCCGTG-3'Biotin
CRAT40-RIP1	5'-GCCCUGCGGUGCCCAGCUCCAUUCAA-3'Biotin
CRAT40-RIP-NC	5'-GCAAUCGAAUGAAAACGUGGAUUCUU-3'Biotin

Table S3. Clinicopathological characteristics of CRC pataients according to CRAT40 expression in TCGA database (n=597).

	N (%)	CRAT40expression		p-value
		high	low	
Gender				0.237
Female	314	165 (52.5%)	149 (47.5%)	
Male	283	135 (47.7%)	148 (52.3%)	
Age (years)				0.574
＜60	167	87 (52.1%)	80 (47.9%)	
≥ 60	430	213 (49.5%)	217 (50.5%)	
Location				0.850
Colon	487	240 (49.3%)	247 (50.7%)	
Rectum	110	53 (48.2%)	57 (51.8%)	
T stage				0.457
T1-T2	124	66 (53.2%)	58 (46.8%)	
T3-T4	473	234 (49.5%)	239 (50.5%)	
N stage				0.795
N0-N1	488	244 (50%)	244 (50%)	
N2-N3	109	56 (51.4%)	53 (48.6%)	
M stage				0.043
M0	459	219 (52%)	237 (48%)	
M1	138	81 (57.4%)	60 (42.6%)	
Stage				0.457
I/II	124	66 (53.2%)	58 (46.8%)	
III/IV	473	234 (49.5%)	239 (50.5%)	
Lymphatic				0.575

No	349	172 (49.3%)	177 (50.7%)	
Yes	248	128 (51.6%)	120 (48.4%)	
BMI				0.094
<27	137	81 (59.1%)	56 (40.9%)	
≥27	158	78 (49.4%)	80 (50.6%)	
Vascular invasion				0.247
No	395	205 (51.9%)	190 (48.1%)	
Yes	122	56 (45.9%)	66 (54.1%)	
Lymphovascular invasion				0.134
No	322	154 (47.8%)	168 (52.2%)	
Yes	215	117 (54.4%)	98 (45.6%)	
Perineural invasion				0.390
No	162	86 (53.1%)	76 (46.9%)	
Yes	56	26 (46.4%)	30 (53.6%)	
Microsatellite instability				0.005
No	100	57 (57.0%)	43 (43.0%)	
Yes	10	1 (10%)	9 (90%)	
Kras mutation				0.425
No	30	14 (46.7%)	16 (53.3%)	
Yes	28	16 (57.1%)	12 (42.9%)	
History of colon polyps				0.229
No	350	180 (51.4%)	170 (48.6%)	
Yes	164	75 (45.7%)	89 (54.3%)	

Table S5. Antibodies used in this study.

Antibody	Source	Application
β-Actin	Cell Signaling Technology	1:2000 for WB
YBX1	Proteintech	1:1000 for WB; 5ug for RIP and CHIP
p65	Proteintech	1:1000 for WB
Phospho-NFκB p65 (Ser536)	Cell Signaling Technology	1:1000 for WB
PCNA	Cell Signaling Technology	1:5000 for IHC
Ki-67	Cell Signaling Technology	1:100 for IHC
Cyclin D1	Cell Signaling Technology	1:50 for IHC
E-Cadherin	Cell Signaling Technology	1:1000 for WB
Bcl-2	Proteintech	1:1000 for WB
Anti-Rabbit IgG (H+L), HRP Conjugate	Cell Signaling Technology	1:3000 for WB
Anti-Mouse IgG (H+L), HRP Conjugate	Cell Signaling Technology	1:3000 for WB
